# Quantifying local ecological knowledge to model historical abundance of long-lived, heavily-exploited fauna

**DOI:** 10.7717/peerj.9494

**Published:** 2020-07-20

**Authors:** Michelle-María Early-Capistrán, Elena Solana-Arellano, F. Alberto Abreu-Grobois, Nemer E. Narchi, Gerardo Garibay-Melo, Jeffrey A. Seminoff, Volker Koch, Andrea Saenz-Arroyo

**Affiliations:** 1 Posgrado en Ciencias del Mar y Limnología, Universidad Nacional Autónoma de México, Mexico City, Mexico; 2 Departamento de Ecología Marina, Centro de Investigación Científica y de Educación Superior de Ensenada, Ensenada, Baja California, Mexico; 3 Instituto de Ciencias del Mar y Limnología – Unidad Académica Mazatlán, Universidad Nacional Autónoma de México, Mazatlán, Sinaloa, Mexico; 4 CoLaboratorio de Oceanografía Social/Centro de Estudios de Geografía Humana, El Colegio de Michoacán - Sede La Piedad, La Piedad, Michoacán, Mexico; 5 Posgrado en Manejo de Ecosistemas de Zonas Áridas, Universidad Autónoma de Baja California, Ensenada, Baja California, Mexico; 6 NOAA – Southwest Fisheries Science Center, La Jolla, CA, USA; 7 Deutsche Gesellschaft für Internationale Zusammenarbeit (GIZ) GmbH, Bonn, Germany, Bonn, Germany; 8 Departamento de Conservación de la Biodiversidad, El Colegio de la Frontera Sur (ECOSUR), San Cristobal de las Casas, Chiapas, Mexico

**Keywords:** Interdisciplinary studies, Ecological modelling, Local Ecological Knowledge (LEK), Sea turtles, Ethnobiology, Conservation, Long-lived fauna, Data-poor fisheries

## Abstract

Deriving robust historical population trends for long-lived species subject to human exploitation is challenging in scenarios where long-term scientific data are scarce or unavailable, as often occurs for species affected by small-scale fisheries and subsistence hunting. The importance of Local Ecological Knowledge (LEK) in data-poor scenarios is increasingly recognized in conservation, both in terms of uncovering historical trends and for engaging community stewardship of historic information. Building on previous work in marine historical ecology and local ecological knowledge, we propose a mixed socio-ecological framework to reliably document and quantify LEK to reconstruct historical population trends. Our method can be adapted by interdisciplinary teams to study various long-lived taxa with a history of human use. We demonstrate the validity of our approach by reconstructing long-term abundance data for the heavily-exploited East Pacific green turtle (*Chelonia mydas*) in Baja California, Mexico, which was driven to near extinction by a largely unregulated fishery from the early 1950s to the 1980s. No scientific baseline abundance data were available for this time-frame because recent biological surveys started in 1995 after all green turtle fisheries in the area were closed. To fill this data gap, we documented LEK among local fishers using ethnographic methods and obtained verified, qualitative data to understand the socio-environmental complexity of the green turtle fishery. We then established an iterative framework to synthesize and quantify LEK using generalized linear models (GLMs) and nonlinear regression (NLR) to generate a standardized, LEK-derived catch-per-unit-effort (CPUE) time-series. CPUE is an index of abundance that is compatible with contemporary scientific survey data. We confirmed the accuracy of LEK-derived CPUE estimates via comparisons with fisheries statistics available for 1962–1982. We then modeled LEK-derived abundance trends prior to 1995 using NLR. Our model established baseline abundance and described historical declines, revealing that the most critical (exponential) decline occurred between 1960 and 1980. This robust integration of LEK data with ecological science is of critical value for conservation and management, as it contributes to a holistic view of a species’ historic and contemporary conservation status.

## Introduction

Assessment of the current population status of long-lived species benefits from a firm understanding of historical baseline abundances (Pauly, 1995). For example, the Internation Union for Conservation of Nature (IUCN) Red List criteria requires abundance trends over three generations. For long-lived species, tracking three generations may necessitate >100 years of data (Seminoff & Shanker, 2008; IUCN, 2019). However, deriving robust historical population trends is challenging when scientific monitoring data are scarce or unavailable (Pauly, 1995; Sáenz-Arroyo et al., 2005; Beaudreau & Levin, 2014). This is further aggravated in data-poor contexts, when a species is impacted by illegal, unreported, or unregulated exploitation. Common data-poor contexts include small-scale fisheries and subsistence hunting (Moller et al., 2004; Duffy et al., 2016; Selgrath, Gergel & Vincent, 2018). This challenging situation has led to increased interest in Local Ecological Knowledge (LEK), including traditional knowledge (TK) of indigenous peoples, to better understand long-term environmental change and human-environment interactions (Johannes, 1981; De Castro et al., 2014; Bao & Drew, 2017; Lee et al., 2018; Barrios-Garrido et al., 2018).

LEK can be defined as place-based empirical knowledge, held by a specific group of people about their surrounding environments and biota (Bélisle et al., 2018). LEK does not require that knowledge-holders be indigenous, nor embedded in a broader shared culture, and thus can be applied to people and communities with relatively short histories of interactions with a specific environment (cf. Narchi et al., 2014). LEK data have been used in combination with official records and historical documentation to reconstruct long-term abundance trends of exploited marine species in multiple contexts (Jackson et al., 2001; Sáenz-Arroyo et al., 2005; Beaudreau & Levin, 2014; Lee et al., 2018). LEK also provides baseline data that fill knowledge gaps which cannot be addressed through natural sciences alone (Mukherjee et al., 2018; Mason et al., 2019). Examples include knowledge of ecological change over broad time-scales (Sáenz-Arroyo et al., 2005; Lee et al., 2018), traditional and local resource use (Johannes, 1981; Barrios-Garrido et al., 2018), and conceptual frameworks for ecological modeling (Ainsworth, 2011; Bélisle et al., 2018). However, clear methodological guidelines, based on robust methods from social and natural sciences, are needed to reliably integrate LEK with scientific ecological data in conservation science (Mukherjee et al., 2018; Young et al., 2018; Moon et al., 2019). This includes developing approaches to collate and validate information from diverse knowledge sources, and forming interdisciplinary teams with expertise appropriate for the methods being used (St. John et al., 2014; Sutherland et al., 2018).

We present a case study of the East Pacific green turtle (*Chelonia mydas*, hereafter green turtle) in Bahía de los Ángeles (BLA), Baja California, Mexico, to demonstrate a novel framework that can be adapted to long-lived, exploited taxa to evaluate abundance trends in data-poor scenarios. We used ethnography to document LEK, and developed an ad hoc epistemological approach to synthesize and quantify LEK data using generalized linear models (GLMs) and nonlinear least squares regression (NLR) to reconstruct long-term *C. mydas* abundance. Our model established baseline abundance, described historical declines, and evaluated how human impacts contributed to current species population status.

The complexity of the green turtle’s life history makes it particularly challenging to evaluate its conservation status. Generation times are up to 50 years, they are highly migratory, and life stages occupy multiple habitats separated by hundreds or thousands of kilometers, often in different countries. Globally, abundance data are skewed towards nesting beaches, which only quantify nesting females (Seminoff & Shanker, 2008; Godley et al., 2010). For the Eastern Pacific population, nesting data have been collected since 1980 at the primary nesting beach in Colola, Michoacán, Mexico (~1,500 km from BLA) (Delgado-Trejo, 2016). However, there are substantial knowledge gaps for foraging habitats, which are critical for several reasons. Foraging habitats include pre-reproductive life stages—which are the most abundant life stages in the population—along with adults of both sexes. Furthermore, foraging habitats are where green turtles spend the majority of their life: juveniles may spend 20 years or more in foraging grounds until reaching maturity, and adults reside at feeding grounds during non-breeding periods (Seminoff, Resendiz & Nichols, 2002; Seminoff & Shanker, 2008; Senko et al., 2019). Thus, expanding data on foraging habitats is of utmost importance for a holistic understanding of population status (Chaloupka et al., 2008; Mazaris et al., 2017; Wildermann et al., 2018).

Green turtles are listed as Endangered by the IUCN and Mexican law as a result of population collapse due to a largely unregulated fishery between the 1950s and 1980s (Diario Oficial de la Federación, 1990; IUCN, 2019; SEMARNAT, 2010). Populations in the Eastern Pacific have increased since the early 2000s thanks to decades of nesting beach protection at Colola starting in the late 1970s, coupled with expanded efforts to limit egg harvests, direct captures, and poaching throughout the species’ range in Mexico (Delgado-Trejo, 2016; Seminoff et al., 2015). These efforts were fortified by the 1990 ban on all sea turtle use in Mexico, which established a legal framework to prevent harvests (Diario Oficial de la Federación, 1990; SEMARNAT, 2010). However, abundance data and long-term trends prior to population collapse are needed to contextualize current population levels (Early-Capistrán et al., 2018; Seminoff et al., 2008).

Starting with an overarching research question (e.g., What was the baseline green turtle abundance, and how did it change over time, before scientific monitoring?), we carried out background research with natural and social science perspectives to gain a broad understanding of the research topic (Crandall et al., 2018). We then used an iterative approach to document LEK through ethnography, and synthesized LEK-data for integration with ecological modeling to provide a consistent long-term time-series of green turtle abundance data that can inform conservation.

## Methods

**Figure 1:**
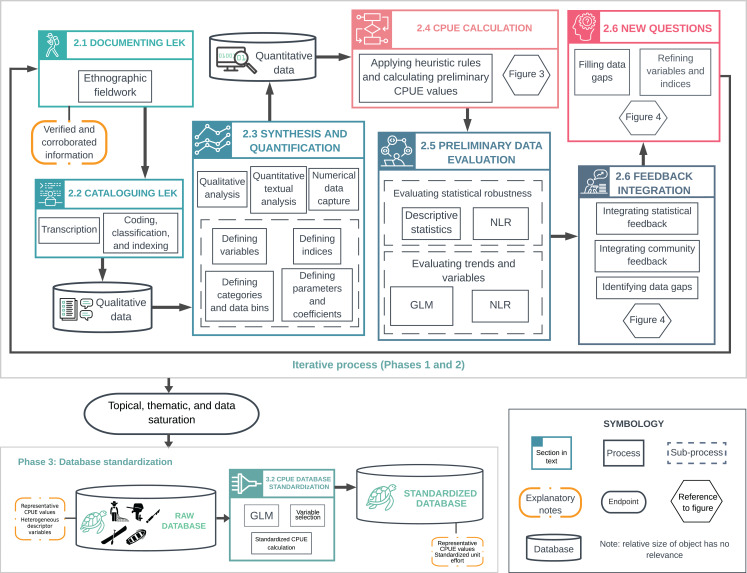
Overview of methodological processes used to document, synthesize and quantify Local Ecological Knowledge (LEK). The upper box illustrates the iterative process described in Phases 1 and 2. This includes LEK documentation and synthesis; analysis with descriptive statistics, generalized linear models (GLMs), and nonlinear regression (NLR); and integration of feedback from statistical analyses and local collaborators. This iterative process was repeated until reaching topical, thematic, and data saturation, and until model fitting did not provide significant new information. The lower box illustrates catch-per-unit-effort (CPUE) data standardization (Phase 3). The raw database (Raw CPUE Database Analysis) contained average, representative CPUE values for a given year, and heterogeneous descriptor variables. We standardized CPUE values using GLMs (CPUE Database Standardization) to (i) remove most of the variation not attributable to changes in abundance, and (ii) generate CPUE values that could be compared over time.

We present a flexible approach for generating green turtle abundance estimates from LEK that can be modified for long-lived species with a history of human use. Our approach consists of four phases: (1) background research and experimental design; (2) an iterative process of LEK documentation, synthesis, and quantification; (3) database standardization and validation; and (4) statistical analysis and modeling of the standardized database (Fig. 1). Interdisciplinary teams can ensure that quality and reliability standards are met across fields (Tengö et al., 2014; St. John et al., 2014; Sutherland et al., 2018). Detailed accounts of methods and tools are available in Supporting Information (henceforth, SI) (Article S1).

### Phase 1: background research and experimental design

#### Study site

To demonstrate our methods, we used the case of the green turtle in Bahía de los Ángeles (BLA), Baja California, Mexico (28°57’6.90"N, 113°33’44.76"W), an index foraging area in the Gulf of California (Seminoff et al., 2003, 2008). We define an index foraging area as a site that (i) has aggregations of turtles in the marine environment that represent a significant proportion of the regional population, and (ii) has been monitored systematically and constantly over a prolonged period of time (>10 years). In-water scientific monitoring in this foraging area began in 1995, after population collapse (Seminoff et al., 2003, 2008). Contemporary scientific monitoring uses catch-per-unit-effort (CPUE) as a measure of abundance (Seminoff et al., 2008).

Green turtles have been a key food source for humans in the arid Baja California peninsula since the earliest phases of human occupation at least 12,000 years ago (cf. Early-Capistrán, 2014). From the late 18th century until the early 1950s, green turtle harvests were primarily subsistence-oriented. Turtles were harpooned from small, wooden canoes propelled with oars or paddles. During the 1960s, the economic and demographic growth along the U.S.-Mexico border led to an increased market for green turtle meat in Mexican border cities. BLA was a key supplier within this trade, and was able to meet demands as the introduction of outboard motors, fiberglass vessels, and set-nets increased cargo volume and catch efficiency. Additionally, improvement of transport and communication infrastructure facilitated market access (Early-Capistrán et al., 2018). The fishery collapsed in the 1970s, green turtle licenses were suspended in 1983 as populations reached dangerously low levels, and all sea turtle fishing in Mexico was banned in 1990 (Márquez, 1996; Seminoff et al., 2008).

#### Background research

This research is part of an on-going collaborative process in the community of BLA which began in 2012 and has included ethnographic and historiographical research related to human–ocean interaction, along with a review of scientific literature (Early-Capistrán et al., 2018). Background research helped define specific research questions, identify challenges in the study design and methods, and develop a general approach for integrating multiple forms of knowledge (Crandall et al., 2018; Early-Capistrán et al., 2018).

Historiographical research situates biological questions in a socio-historical context, providing information on a species’ past abundance which can be correlated with time-frames, social processes, and management regimes (Article S1) (Crandall et al., 2018; Sáenz-Arroyo et al., 2005). Historiographical research helped us understand human-green turtle interactions in BLA over the past three centuries, identify the early 1960s as a period when human impacts precipitated a major decline in green turtle abundance in BLA, and establish the early 1950s as a time-frame for reconstructing baseline abundance before large-scale commercial exploitation (Early-Capistrán et al., 2018).

Long-term collaboration with the community of BLA was fundamental for previously establishing the rapport and working trust necessary to conduct transdisciplinary research. Long-term engagement has also helped us acquire sensitivity to the cultural context, gain an understanding of social conditions, and gather locally-relevant information to define research questions and design (Bernard, 2011; Crandall et al., 2018). We also established a network of local collaborators, whom we define as knowledgeable community members willing to share their knowledge and expertise (Crandall et al., 2018). Due to the fact that ecological knowledge is differentially acquired by social actors, we constructed a heterogeneous network of social actors with diverse types of knowledge that, when nested together, construct the ecological knowledge around green turtle abundance (cf. Brown, 2010).

#### Experimental design

##### Qualitative methodology

Ethnography was our primary data-gathering methodology. This holistic approach to the study of social systems uses a varied toolkit to generate both qualitative and quantitative data (Table 1; Article S1; Table S1). Ethnography requires rapport, sensitivity to the cultural context, and developing an understanding of the social system on its own terms. Data are gathered broadly over topic areas and new questions are developed continuously (Bernard, 2011; Early-Capistrán et al., 2018). Ethnography also helps identify biases by analyzing data within a social and historical context (Drury, Homewood & Randall, 2011). Ethnographic data are systematized, cross-referenced, verified, and subject to analysis and meta-analysis (Bernard, 2011).

**Table 1:** Methods used for data collection during ethnographic field work. Sources: Bernard (2011); Crandall et al. (2018); Early-Capistrán et al. (2018).

Method	Definition	Example of applications	Practical implications
Participant observation	Studying a social group through a combination of direct observation and immersion in group activities as an active participant	Participating in and documenting sport-fishing trips led by former green turtle fishers	All observations are compiled in field notes and journals, including, but not limited to research topics
Informal interviews	Interviews without structure or control, often conversations held during the course of fieldwork	Conversations with fishers or their family members recorded in written notes	Recorded in field notes and field journals
Semi-structured interviews	Interview based on a flexible list of written questions or topics that need to be covered. The interviewer maintains discretion to follow new leads	Contributors were interviewed using an interview guide with recurring topics focused on the green turtle fishery	Recorded in audio or video with the collaborators’ consent
In-depth interviews	Aimed at obtaining detailed understanding of the topic of interest. Participants can communicate more freely and provide more detailed descriptions than with semi-structured interviews	Experts and key local collaborators were interviewed in-depth on specific topics related to green turtle fishing or abundance (e.g.: fishing gear, green turtle commerce, etc.)	Recorded in audio or video with the collaborators’ consent
Focus groups	Moderated discussions with small groups (<10 people) on a particular topic	Focus group discussions with members of a fishing crew to discuss how green turtle abundance changed over the course of their careers	Recorded in audio or video with the collaborators’ consent
Oral histories	In-depth interviews about life stories, experiences, and eyewitness accounts	Interviewing experts on their life history and their experience as green turtle fishers	Recorded in audio or video with the collaborators’ consent
Participatory mapping	Contributors draw maps, locate key places on maps, or locate key sites together with researchers	Visiting key green turtle fishing spots and recording coordinates with GPS	Recorded in notes, digital maps, GIS or printed maps
Social network analysis	Identifying the structure of social relations	Documenting kinship and work relations among green turtle fishers and merchants	Recorded in notes and graphs
Discourse analysis	Analysis of communicative content and structure focused on how meaning is constructed and how power functions in a society	Analyzing discourse on regulation or conservation to identify biases that could affect how fishers report on turtle catches	Analysis of ethnographic materials; feedback integrated into new questions

We chose ethnography because (i) the high degree of socio-environmental complexity required detailed information on diverse topics; (ii) sea turtle fishing is currently illegal in Mexico and its inquiry requires working trust, long-term engagement, and confidentiality; and (iii) ethnography provides more detailed and reliable information on sensitive issues than is provided by questionnaires (Drury, Homewood & Randall, 2011; St. John et al., 2014). Research was designed in compliance with the ethical guidelines of the International Society of Ethnobiology (Articles S1 and S2) (International Society of Ethnobiology, 2006) and approved by the Bioethics Committee of the Centro de Investigación Científica y de Educación Superior de Ensenada (Approval Number 2S.3.1).

We defined three social groups within the community and documented their knowledge. Fishers who participated in the legal green turtle fishery before 1990 (henceforth, turtle fishers) constituted the target population and provided the majority of specialized LEK related to human-turtle interaction. This group was the main focus of ethnographic research and contributed the majority of the qualitative and numerical data. Key local collaborators—defined as community members with expertise in particular topics—provided important complementary and contextual information on topics such as local history, commerce, or foodways, among others. Finally, we gathered additional complementary data from members of the community at large (henceforth, community members), including fishers’ families, green turtle merchants, local authorities, commercial and sport fishers, and conservation workers, to understand and incorporate multiple perspectives. Methods and sample sizes used for each of these groups are discussed in detail in “Documenting LEK”.

We designed flexible interview guides for use in semi-structured and in-depth interviews based on previous ethnographic research on sea turtle use in BLA (Early-Capistrán et al., 2018). Interviewers M.M.E.C. and G.G.M. used these guides as a roadmap for the interviews, allowing respondents to be thorough and make associations between questions, and to include new topics and questions according to interview progress (cf. De Castro et al., 2014). Interview guides covered five main topic areas: (1) biographical profile and career history; (2) sea turtle consumption and commerce; (3) trends in sea turtle captures and sizes; (4) spatial distribution of sea turtle fishing; and (5) fishing effort and technology (Box 1). To prompt recollection of dates, questions were associated with important events in local collaborators’ lives (Article S1). We piloted questions with local fishers outside the target population (*n*_pilot_ = 2) and constantly refined the questions to ensure that they were locally contextualized and elicited meaningful answers (Bernard, 2011; Drury, Homewood & Randall, 2011; Young et al., 2018).

**Box 1:** Primary topic areas in interview guides.

1. Biographical data and career history
Year of birth
Years in the community
Years as a fisher
Years in the green turtle fishery
Crew members and fishing merchants with whom they worked
2. Sea turtle consumption and commerce
Domestic sea turtle consumption dynamics (before 1990 ban)
Market dynamics for sea turtle sale (how, where, and how often turtles were shipped)
Commercial dynamics (how turtles were sold, prices, working relationships, etc.)
3. Sea turtle catches and sizes
Maximum and minimum catches
Frequency of aggregations and large catches
Average catches
Perceived changes in abundance
Size distribution (maximum and mode sizes, frequency of catching large turtles)
Sea turtle ethnobiology (effects of seasonality, tides, green turtle behavior, etc.)
4. Spatial distribution of fishing
Frequently used fishing grounds
Hot-spot and aggregation dynamics
Changes in use of fishing grounds across time
Distances and travel times to fishing grounds
5. Fishing effort and technology
Use and efficiency of different gear types/gear designs
Use of different vessels
Use of different propulsion systems

##### Reconstructing green turtle abundance through collective knowledge

Defining an approach to estimate green turtle abundance based on CPUE was a key challenge. Although CPUE is a crude measure of changes in exploited populations (López-Castro et al., 2010), we used it because (i) it is the only available metric of current abundance and (ii) CPUE is an accepted proxy for abundance for IUCN Red Listing (IUCN, 2019; O’Donnell, Pajaro & Vincent, 2010).

Adequate assessment of CPUE as a measure of abundance requires detailed understanding of the fishery and the variables that affected it (Moller et al., 2004). The skilled turtle fishers of BLA almost always targeted high-density locations (hot-spots) and aggregations, and thus maximized CPUE by optimizing fishing patterns based on empirical knowledge of environmental conditions and green turtle behavior (Early-Capistrán et al., 2018). Consequently, turtle fishers’ expertise allowed for high CPUE events over time despite declining overall abundance (hyper-stability), underscoring the need to (i) account for this non-random search behavior and (ii) understand central CPUE trends rather than exceptional catches (Article S1; Fig. S1) (Anticamara et al., 2011; Early-Capistrán, 2014; Maunder & Punt, 2004; Selgrath et al., 2018; Walters, 2003).

This scenario is challenging, as (i) interviewees’ memory of “typical” events may be less accurate than that of salient events and (ii) high variability in CPUE and changes in fishing efficiency can mask overall abundance trends (Maunder & Punt, 2004; De Damasio et al., 2015; Sáenz-Arroyo & Revollo-Fernández, 2016). Thus, we designed our methodology to calculate CPUE based on multiple sources rather than individual recollections. We also aimed to identify and account for sources of variation in CPUE that could bias proportionality with abundance, and to construct adequate proxies for variables such as spatial distribution of fishing, differences in gear types, and changes in fleet conditions (Walters, 2003; Maunder & Punt, 2004; Anticamara et al., 2011; Selgrath, Gergel & Vincent, 2018).

**Figure 2:**
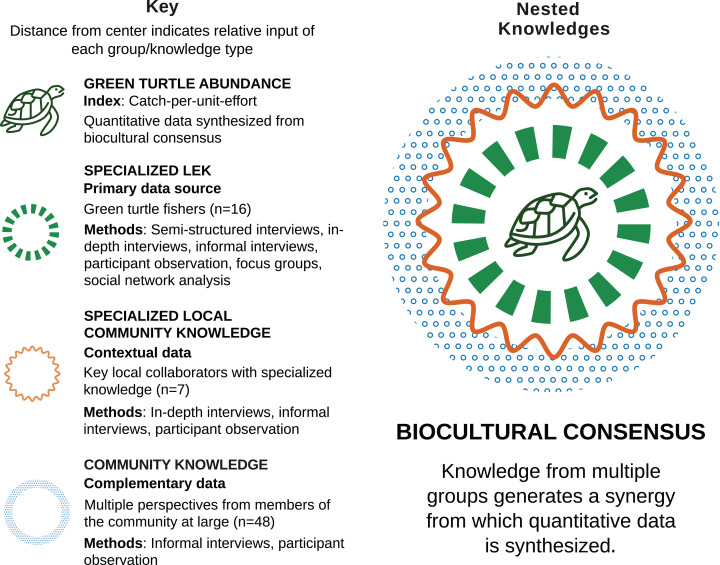
Biocultural consensus as a synergy of interconnected knowledge (adapted from Brown, 2010). Biocultural consensus was constructed with multiple and complementary contents and knowledge types from three different social groups. Sample sizes, ethnographic methods, and interview methods used with each group are provided. The target population of turtle fishers were the group with which we collaborated most intensively and which provided the majority of LEK, as well as qualitative and numerical data. Turtle fishers’ knowledge was complemented with knowledge from of key local collaborators and community members. Biocultural consensus helped build conceptual frameworks for modeling, establish limits and assumptions, estimate model parameters, and validate model outputs.

We approached CPUE as a component of a holistic dataset on human-environment interaction, and aimed to synthesize quantitative values on the basis of biocultural consensus, which we define as the pooling of information for evaluating shared environmental perceptions constructed by the summation of individual, community, specialist, and holistic types of knowledge. Biocultural consensus is a synergistic, interconnected set of contents and types of knowledge (c.f. Brown, 2010) in which the resulting knowledge is greater than sum of its parts. In this case, we used knowledge from all three social groups (turtle fishers, key local collaborators, and community members) as inputs for constructing biocultural consensus. Our ethnographic research was primarily focused on turtle fishers, who provided the majority of qualitative and numerical data, as well as specialized LEK related to human-turtle interaction. Key local collaborators and community members provided contextual and complementary data (Fig. 2). Biocultural consensus helped build conceptual frameworks for modeling, establish limits and assumptions, estimate model parameters, and validate model outputs (Bélisle et al., 2018).

As the primary response variable, we aimed to calculate representative values of CPUE during a specific year with the initial definition:

(1) $${\rm CPUE\;=\; number\; of\; turtles\; caught/unit\;effort}$$

For initial inquiry, we used the working definition of one unit effort as one night of fishing (∼12 h) with either a harpoon or a set-net (Maunder & Punt, 2004). We continually refined and updated this definition as we gained further information on fishing technology, effort, and efficiency through the iterative feedback process between qualitative data, NLR, and GLMs (Phase 2). We then standardized CPUE estimates to account for differences in gears and changes in efficiency (Phase 3). As the final result of the iterative feedback process, we obtained standardized, representative mean CPUE values for a specific year, based on biocultural consensus of green turtle captures.

##### Quantitative methods

Throughout the iterative process, we used descriptive statistics for exploratory data analysis and to identify outliers (Zar, 2014). We used NLR to describe CPUE trends over time and GLMs to identify significant predictor variables. We also integrated residual analysis to ensure that model assumptions were met and to evaluate goodness of fit and robustness. We ensured that residuals met the assumptions of zero mean, normal distribution, homoscedasticity, and independence (*e_i_* ~ *N*(0, σ^2^)) (Table 2; Article S1) (Maunder & Punt, 2004; Ritz & Streibig, 2008). In response to residual auto-correlation found during preliminary phases and exploratory data analysis, as is common with time series data, we integrated residual correlation structures to GLMs with residual auto-correlation during the final stages of standardization (Zuur, 2009). All models reported in the Results meet the assumptions for robust residuals according to the criteria described in Table 2 (see also Article S1).

**Table 2:** Tools and criteria for the model fitting and selection processes. Throughout the iterative process, we used nonlinear regression to describe catch-per-unit-effort trends over time, and generalized linear models to identify significant predictor variables. Residual analyses were used to ensure that model assumptions were met, and to evaluate goodness of fit and robustness.

Process	Software	Model selection criteria	Residual analyses
Preliminary model selection and starting values	LABFit 7.2.49	*R*^2^ value	–
Nonlinear regression (NLR)	R 3.4 (*nlstools* and *easynls* package)	*R*^2^ value Robust residuals: *e_i_* ~ *N*(0, σ^2^)	Normality: Shapiro–Wilk test, *p* > 0.05 Mean = 0: *t*-test, *p* > 0.05 Homogeneity of variance: Levene’s test, *p* > 0.05 Randomness: runs test, *p* > 0.05 Auto-correlation: Pearson correlation test (residuals vs. lagged residuals), *p* > 0.05 (i.e., H_o_: ρ = 0, *H*_a_: ρ ≠ 0)
Generalized linear model (GLM)	R 3.4 (*nlme*, *lmtest* and *car* packages)	Significant predictor variables (*p* < 0.05) *D*^2^ value Low relative AIC Robust residuals: *e_i_* ~ *N*(0, σ^2^)	Normality: Shapiro–Wilk test, *p* > 0.05 Mean = 0: *t*-test, *p* > 0.05 Homogeneity of variance: Levene’s test, *p* > 0.05 Randomness: runs test, *p* > 0.05 Auto-correlation: Durbin–Watson test, *p* > 0.05

It should be noted that the statistical treatment is applied to the data series synthesized from biocultural consensus. We used all available information to amass a year by year remembrance of turtle captures by combining fishers’ knowledge with that of key local collaborators and community members. Thus, our synthesized data is not derived directly from the individual, yearly recollections of specific fishers, but instead are the result of collectively generated and corroborated knowledge. Likewise, statistical analyses were not conducted in relation to the social groups themselves (aside from simple demographic description), but rather to the quantified data synthesized from their collective knowledge, which included sea turtle captures as well as descriptor variables, coefficients, and indices (Table 3).

**Table 3:** Variables, coefficients, and indices.

Variable or coefficient	Type	Index	Source
Year of birth	Numerical	Date	Standard question in interviews
Dates working in the green turtle fishery	Range	Interval of dates	Standard question in interviews
Experience in the green turtle fishery	Ordinal	1 = 1–5 years 2 = 6–10 years 3 = 11–15 years	Binned from dates working in the fishery
Generation	Categorical	1 = Fishers who worked in commercial development and commercial fishing stages	Category of cohorts of fishers defined based on the fishery stages in which the contributor worked
		2 = Fishers who worked during the collapse stage	
		3 = Fishers who worked through all stages	
Fishery stage	Categorical	1 = Commercial development	Defined based on qualitative data on the fishery
		2 = Commercial fishing (harpoon)	
		3 = Commercial fishing (nets)	
		4 = Collapse	
Year	Numerical	Date for which the average CPUE is being described	Obtained directly from interviews (numerical value) or calculated based on heuristic rules (details in S.I.)
Fishing gear	Ordinal	1 = Harpoon	Binned from interviews or inferred based on heuristic rules
		2 = Short set-net (~100 m)	
		3 = Long set-net (~200 m)	
Harpooner skill coefficient	Percentage	Percentage of success (50–99%)^a^	Obtained from interview data and assigned to contributors based on social network analysis
Number of nets	Numerical	Number of nets used^b^	Obtained directly from interviews or inferred based on heuristic rules
Vessel type	Ordinal	Type of vessel used 1 = Wooden canoe (12–15 ft length)	Binned from interviews or inferred based on heuristic rules
		2 = Fiberglass skiff (20–22 ft length)	
		3 = Boat (variable length)	
Vessel capacity	Ordinal	Gross vessel tonnage	Binned from interviews or inferred based on heuristic rules
		1 = Less than 1 ton	
		2 = 1–1.5 tons	
		3 = Greater than 1.5 tons	
Propulsion^c^	Categorical	1 = Oars 2 = Motor (5–10 horse-power) 3 = Motor (15–40 horse-power)	Obtained directly from interviews or inferred based on heuristic rules
Trip duration^c^	Numerical or interval	Number of days between leaving port and returning with a catch of turtles at vessel capacity Minimum limit: 1 day Maximum limit: 10 days	Obtained directly from interviews or inferred based on heuristic rules (S.I., Eqn. S1, S2)
Fishing time	Numerical	Number of nights spent fishing on a trip of regular duration	Obtained directly from interviews or inferred based on heuristic rules (S.I., Eqn. S1, S2)
Average CPUE	Numerical	Average number of turtles caught in one night during a specific year	Obtained directly from interviews (numerical value) or calculated based on heuristic rules

**Notes:**

a Not assigned to captures with nets.

b Not assigned to harpoon captures.

c Proxies for spatial distribution of fishing.

### Phase 2: recording, synthesizing, and quantifying LEK

#### Documenting LEK

M.M.E.C. and G.G.M. compiled ethnographic data in BLA over three field seasons (spring 2017, summer 2017, and spring 2018) and 57 working days. We obtained oral informed consent from all participants prior to the start of interviews, and recorded interviews in audio or video and took technical photographs when possible (Article S1; Tables S2 and S3) (International Society of Ethnobiology, 2006). We chose oral consent as it was not deemed culturally appropriate to ask participants to sign a consent document and because some participants were not comfortable with written language (International Society of Ethnobiology, 2006; Wedemeyer-Strombel et al., 2019). We conducted all interviews in Spanish—our primary language and that of the collaborators—and transcribed recorded interviews in digital format (.txt). We also compiled field journals in digital format (.txt), recording all observations in detail (Article S1).

We validated ethnographic data through triangulation among (i) participants (e.g., data were independently corroborated and verified by multiple local collaborators), (ii) sources (e.g., documents, photographs, scientific literature, etc.), and/or (iii) methods (e.g, interviews, archive research, etc.). Once processed, we member-checked data for reliability by asking local collaborators from all groups if our themes or categories were locally relevant and congruent. We also asked local collaborators to identify data gaps, and inquired if overall accounts and processes were described in a manner that was realistic and accurate (Creswell & Miller, 2000; Tengö et al., 2014). Prolonged engagement in the field allowed us to compare interview data with observations, and helped build trust so that participants were comfortable disclosing information, increasing reliability in responses (Bernard, 2011).

We identified turtle fishers using a deliberate hierarchical sampling method (Bernard, 2011), Turtle fishers are a small group of the oldest fishers in the community, between 55 and 85 years of age (*N*_fishers_ = 17). We interviewed 94% of turtle fishers, as one fisher chose not to participate. All fishers in the population and sample were men. With this target group, we continuously carried out participant observation, and conducted 17 semi-structured interviews (at least one per person), along with 27 informal interviews. Within this target population, we identified a subset of seven expert LEK holders, which we defined as turtle fishers recognized as experts by at least two peers, and whose empirical and specialized knowledge can be used as a basis for inferences and assessments about their surrounding environments and biota (cf. Bélisle et al., 2018). With the group of expert LEK holders, along with the aforementioned methods, we conducted seven in-depth interviews and one focus group discussion to gather specialized data (Tengö et al., 2014).

We identified key local collaborators (*n*_klc_ = 7) through purposive and respondent-driven sampling (Bernard, 2011). Key local collaborators were primarily older (>63: 71%) and included women (43%) and men (57%). We continuously carried out participant observation with this group, and conducted four in-depth interviews and 23 informal interviews. Topics included: local history, economy, commerce, and foodways; marine and terrestrial ethnobiology and conservation; and commercial and sport fishing, among others, which provided valuable information for situating green turtle fishing within a broader socio-ecological context (Crandall et al., 2018).

We selected local collaborators from the community at large (*n*_cm_ = 48) through a combination of cluster sampling and self-selection (Bernard, 2011). They represented ~8% of the population of BLA and included women (42%) and men (58%). Ages ranged from 18 to 93, with young (18–39: 35%), middle-aged (40–62: 37%), and older (>63: 28%) participants. While we did not inquire about income given local social taboos, local collaborators came from across all class strata with schooling varying from individuals without formal schooling to graduate degree holders. The group included both long-term residents (89%) and short-term residents (11%) such as conservation workers and government employes. This diverse group provided a broad view of perspectives and topics to complement and contextualize information from the target population of turtle fishers. With this group, we continuously carried out participant observation and conducted 72 informal interviews.

#### Cataloguing LEK

We processed and coded all field journals and interview transcriptions following a standardized protocol. We used footnotes to separate observations from analysis, and for cross-referencing. Cryptic indicators ensured local collaborators’ anonymity (Bernard, 2011). We used cultural material codes (Murdock et al., 2008) to categorize ethnographic data, with customized codes for topics and themes specific to this research. We indexed text entries using hashtags (#) to mark relevant topics (e.g., #fishing_gear), including ordinal codes (e.g., #max_cpue; #min_cpue) to classify information for data-binning (Article S1; see Table S4 for an example field journal entry). Along with data compiled in the 2017 and 2018 field seasons, we coded and indexed ethnographic materials collected since 2012 for integration into the qualitative database (Article S1; Tables S2 and S3). Coding allowed us to break down qualitative data into analytical variables and raw values (Strauss & Corbin, 1994). Digital files allowed for analyzing large volumes of information by facilitating topic-specific searches, generating a corroborated, systematized, and cross-referenced qualitative database (Bernard, 2011).

#### Synthesizing and quantifying LEK

##### Qualitative analyses

We used qualitative textual analysis and discourse analysis to decipher the cultural, historical, and political dimensions of the research topic; to identify potential sources of bias; and to understand categories, processes, and connections (Crandall et al., 2018) (Table S1). We captured raw numerical data from interviews (Article S1; Table S4), and used Quantitative Textual Analysis tools in R 3.4 (*wordcloud*, *tm*, and *SnowBallC* packages) to identify themes and patterns over large volumes of text, for a general overview (Bernard, 2011; R Core Team, 2019) (Article S1; Figs. S2 and S3). These themes helped us to identify potential descriptor variables, indices, and topics for inquiry.

##### Quantifying LEK data

We defined explanatory variables for CPUE based on qualitative data (Table 3). We generated initial indices for each variable based on the degree of detail and variation observed in interview responses, and defined standardization and binning procedures (Fig. 1).

We established four stages for the BLA green turtle fishery based on fishery landing statistics and previous research (Early-Capistrán et al., 2018; Selgrath, Gergel & Vincent, 2018): (1) commercial development; (2) commercial fishing (harpoons); (3) commercial fishing (nets); and (4) collapse (Table 4). Qualitative data allowed for inferring that (i) fishing technology across the fleet was similar within each stage; (ii) at all stages, fishers would make trips of varying duration until reaching vessel capacity or exhausting food and water supplies; and, thus, (iii) CPUE could be calculated based on the knowledge of fisheries stages, trip duration, fishing gear type, displacement time, and vessel capacity (Article S1). This framework allowed us to (i) bin data and standardize variations in expertise and response terms, (ii) systematically complement the knowledge of less experienced fishers with that of expert LEK holders, and (iii) account for changes in fishing technology, effort, and efficiency over time (cf. Maunder & Punt, 2004).

**Table 4:** Fishery stages and characteristics.

	Commercial development (1950–1959)	Commercial fishing (harpoons) (1960–1965)	Commercial fishing (nets) (1966–1972)	Collapse (1974–1982)
General characteristics	First years of the commercial fishery, with limited technology and fishing effort	Intense growth in demand leads to declining captures	Increasing fishing effort and efficiency, declining captures	Commercial collapse. Species abundance near extinction.
Regulation	Unregulated	Unregulated	Limited regulation: minimum size, permit restrictions, seasonal bans Temporary ban (1971)	Highly regulated: minimum size, permit restrictions, seasonal bans, nesting beach protection (1980-present) Green turtle licenses suspended (1983)
Gear type	Harpoons	Harpoons	Set-nets	Set-nets
Fleet conditions	Wooden canoes Oars or paddles	Wooden canoes 5–10 horse-power outboard motors	Canoes or skiffs 5–10 horse-power outboard motors	Fiberglass skiffs 15–45 horse-power outboard motors
Spatial distribution of fishing^a^	Overnight trips close to port are frequent	Motors allow faster displacement to farther fishing grounds Occasional trips >50 nautical miles	Trips >50 nautical miles are frequent Expeditions >100 nautical miles are frequent (canoes or skiffs off-loading to boats)	Trips >50 nautical miles are frequent
Size distribution^b^	Turtles ~150 kg caught frequently (spans of weeks/months) Mode weight: 50 kg	Turtles ~150 kg caught frequently (spans of weeks/months) Mode weight: 50 kg	Turtles 100–150 kg caught occasionally (spans of seasons/years) Mode weight: 50 kg	Turtles 100–150 kg caught rarely (spans of years) Mode weight: 50 kg
Fishing efficiency	Low	Low/Moderate	Moderate	High
Fishing effort	Low	High	High	Low
Commercial demand	Moderate	High	High/moderate	Moderate
Profitability	High	High	High/Declining	Not profitable

**Notes:**

a Throughout the chronology, spatial distribution of fishing was highly variable due to the targeting of hot-spots and variations in the seasonal distribution of turtles.

b Size distribution was highly variable throughout the chronology.

Characteristics from qualitative LEK and Early-Capistrán et al. (2018), Márquez (1996), and Seminoff et al. (2008).

We generated digital (.txt) files to summarize categorical, ordinal, and numerical data for each fisher (Article S1; Table S5). Using social network analysis (Bernard, 2011), we situated each fisher in relation to their fishing crew and extended family (Table 1). Ethnographic and LEK data provided us with numerical anchor values and limits for variables during each stage (Bélisle et al., 2018) (Article S1).

#### CPUE calculation and preliminary database generation

**Figure 3:**
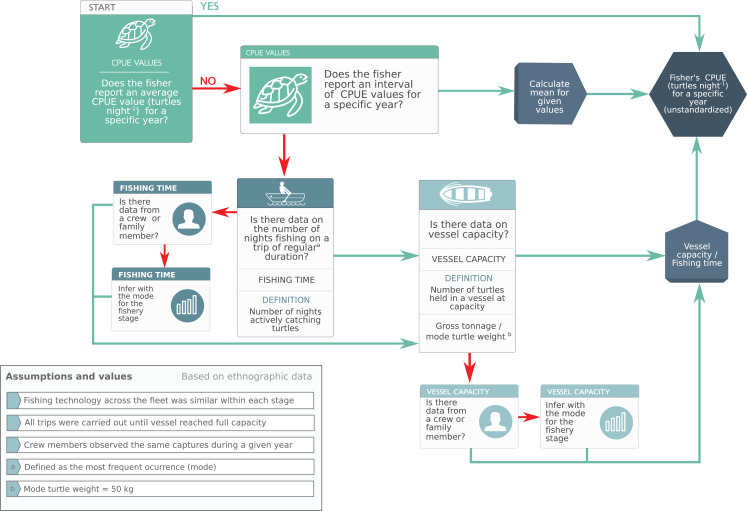
Heuristic rules used to make systematic inferences based on expert knowledge to calculate raw catch-per-unit-effort values. We used heuristic rules to make systematic inferences based on the knowledge of expert turtle fishers (Phase 2, “CPUE Calculation and Preliminary Database Generation”). This framework allowed us to reduce individual cognitive bias by (i) complementing the knowledge of less experienced fishers with that of experts, and (ii) calculating a central tendency based on collectively-generated knowledge and biocultural consensus.

To deal with variability, we used heuristic rules to make systematic inferences based on the knowledge of expert LEK holders (Fig. 3). This framework allowed us to calculate a central tendency based on collectively-generated knowledge and biocultural consensus rather than individual recollection, thus reducing individual cognitive bias (Article S1).

We converted captures reported by weight to number of turtles by dividing vessel capacity by mode of turtle mass (50 kg) reported by fishers and corroborated with monitoring data (Early-Capistrán et al., 2018) (Article S1). While turtle size was highly variable and likely declined in response to increasing fishing effort (Table 4), mixed juvenile/adult foraging groups with a slight juvenile bias—such as BLA, where ~56% of individuals are juveniles (Seminoff et al., 2003)—are present in green turtle foraging habitats worldwide (Seminoff et al., 2015). Thus, we consider our assumption regarding size distribution to be adequate given the nature of the data (Table 4; Article S1).

#### Preliminary data evaluation

We estimated CPUE and descriptor variables through an iterative process. We stored data in .csv format and carried out all analyses in *R 3.4* unless otherwise specified (R Core Team, 2019). We analyzed descriptive statistics to evaluate statistical robustness by checking data distribution, evaluating normality (Shapiro–Wilk *p* > 0.05), and identifying outliers (±2 SD) (Zar, 2014). Each CPUE data point was linked to a summary of qualitative and numerical data for a specific collaborator, and outlying data were contextualized and evaluated (Article S1; Table S5). Over the course of the iterative process, we discarded three CPUE values from fishers who (i) had less than 1 year of experience and (ii) were very young (10–13 years of age) when they captured turtles. During interviews, these fishers recognized that they had limited recollection of events and did not have the experience necessary to provide precise data. Statistical analysis confirmed that CPUE values provided by this group were outliers (±2 SD).

To evaluate CPUE trends, we converted values for the independent variable “year” to serial form in all analyses. We used *LABFit 7.2.49* to identify five preliminary models with best fit and their respective starting values. We then ran NLR (*nlstools*, *easynls, dplyr, car*, and *DescTools* packages; Data and Code) to choose the model that best described the data, and evaluated residuals (Table 2). We ran NLR at each round of the iterative process to (i) evaluate the general behavior and performance of the data, (ii) identify outlier effects in residual analysis, and (iii) evaluate if the process was robust to these effects (Baty et al., 2015; Ritz & Streibig, 2008). An exponential decay model consistently showed the best fit, with the form

(2) $$Y {\sim} {\rm{\alpha}} \cdot e^{({\rm{\beta}}x)}$$

where *Y* is the response variable, CPUE; α is a constant (intercept); β is an instantaneous rate of change in the response variable (slope); and *x* is the independent variable “year”.

We used GLMs with a link function for Gaussian distributions to identify significant predictor variables for CPUE (*nlme, dplyr, car* and *DescTools* packages), using log-transformed values if the CPUE distribution was non-normal (Zar, 2014). We ran backward-stepping models until we obtained a model with significant effects, a high percentage of explained deviance (*D*^2^), a relatively low Akaike Information Criterion (AIC), and robust residuals (Table 2) (cf. Maunder & Punt, 2004).

**Figure 4:**
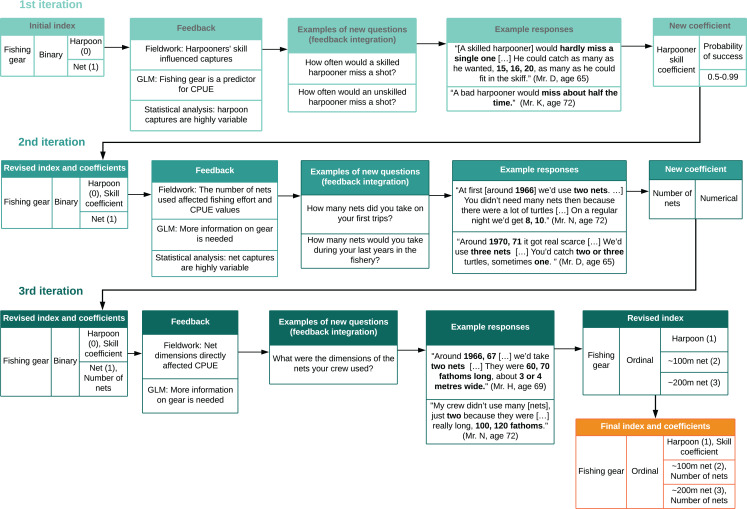
Cyclical process of index design and feedback integration. We revised indices and coefficients based on a cyclical process which used feedback from interviews, statistical analysis, and generalized linear models (GLMs) to design new questions. This was repeated for each variable throughout Phase 2. Bold type shows numerical data from interviews.

We ran a total of 36 NLR and 54 GLMs on five sequential working databases. The first three databases each corresponded to one round of the methodological cycle (Fig. 1). With each round, the working databases were updated and superseded as we incorporated new data, variables, indices, analyses, and data cleaning processes (Figs. 1, 3 and 4). The last two databases consisted of the final raw database—with LEK-derived CPUE values and heterogeneous variables for unit effort—and the standardized database with mean standardized CPUE values for each year (Data and Code). By integrating these analyses into the cyclical process, we are confident that we adequately identified confounding variables and sources of variation not attributable to changes in abundance (Hilborn & Walters, 1992).

#### Feedback integration

We integrated model-fitting feedback by identifying which variables and indices required further information or could be improved (Fig. 4). We integrated feedback from community members during subsequent visits to the field by sharing preliminary results and model outputs with them through narrative description, and asking for collaborators’ perspectives on validity and consistency (Bélisle et al., 2018). Local collaborators also identified gaps and provided further information (Huntington, 2000; Tengö et al., 2014). We then designed new questions based on feedback and repeated these procedures with each variable (Figs. 1 and 4).

We repeated the cyclical process of data gathering, synthesis, and quantification until reaching topical saturation (similar instances were repeated and no additional data were found with which to develop new properties), thematic saturation (additional data did not produce new emerging themes), data saturation (new data repeated what was expressed in previous data) (Saunders et al., 2018), and until model fitting did not provide significant new information.

Time frames required to reach saturation are extensive, in the order of months or years. Ethnographic fieldwork generally requires a year or more, given the extensive time required to establish rapport, obtain working knowledge and understanding of the cultural context, and to be able to ask good questions and obtain good answers (Bernard, 2011). The interview process to elicit the data presented in this article represented 57 working days over three field seasons (spring 2017, summer 2017, and spring 2018). While it may seem a rather short timeframe, it must be said that two of the authors, M.M.E.C. and G.G.M., have been conducting ethnographic work in the community since the summer of 2012, making seven trips to the region with a mean duration of 27 days, conducting a total of 378 interviews to date (Tables S2 and S3), and maintaining contact and communication with community members between field seasons. Long-term continuous interaction has allowed rapport for intelligible dialog among researchers and local community members in ways that enable elicitation of trustworthy data.

### Phase 3: database standardization and validation

#### Raw CPUE database analysis

The result of the methodological cycle was a final, LEK-derived CPUE database with heterogeneous variables for unit effort (raw database) (Fig. 1). We carried out descriptive statistical analysis, NLR, and GLM analysis to evaluate the data and define standardization procedures as described in “Preliminary Data Evaluation”.

#### CPUE database standardization

We standardized CPUE to (i) remove most of the variation not attributable to changes in abundance by accounting for variables such as gears, fleet characteristics, fishers’ experience, etc.; and (ii) generate CPUE values that could be compared over time (Hilborn & Walters, 1992; Maunder & Punt, 2004). To choose predictor variables for standardization, we ran GLMs (*nlme*, *car*, *dplyr*, and *DescTools* packages; Data and Code) with log-transformed CPUE values and a residual correlation structure based on an auto-regressive model of order 1 (*AR-1*) structured by the variable “year” (Zuur, 2009). We chose predictor variables for standardization using models with significant effects, high percentage of explained deviance (*D*^2^), relatively low Akaike Information Criterion (AIC), and robust residuals (Table 2) (cf. Maunder & Punt, 2004).

We generated detailed definitions of unit effort based on these analyses, in order to obtain comparable values for turtles caught in one night. While fishers generally worked from dusk to dawn, fishing times on any given night with either gear type could be variable. For modeling purposes, we simplified values to 12 h blocks which reflect the vast majority of fishing effort (Article S1).

For set-nets, we standardized unit effort to approximate ecological monitoring data (100 m net soaking for 12 h) (Koch, 2013; Seminoff et al., 2003):

(3) $$C_{\rm st} = (t \cdot R) / (n_r \cdot R\cdot 12\;{\rm h})$$

where *C*_st_ is a standardized, representative value of CPUE during a specific year (turtles 12 h^−1^); *t* is the number of turtles caught (turtles); and *n*_*r*_ is the number of 100 m nets (no units). *R* is net length (in multiples of 100 m), simplified to short (~100 m = *R*) or long (~200 m = 2*R*) (Table 3). Soaking time is 12 h.

For harpoon captures, we assigned a skill coefficient (*s*, percentage of success) (Table 3) to each harpooner through social network analysis (Table 1), based on colleagues’ assessment, such that:

(4) $$C_{\rm st} = t\cdot {\rm s}^{-1}\cdot 12\;{\rm h}^{-1}$$

The current ban on sea turtle fishing does not allow us to test for differences in susceptibility to fishing gears. Harpoons and nets were not used simultaneously by any given fisher, and both were used over a roughly equivalent number of hours per night. Thus, we considered these values to be adequately standardized given the nature of the data. For years with multiple CPUE values, we calculated the mean after standardization (Article S1; Figs. S4 and S5).

#### Evaluating statistical robustness

We evaluated reliability through comparison with green turtle fishery statistics for BLA (annual landings in tons, 1962–1982) (Márquez cited in Seminoff et al. (2008)). CPUE and total landings are both crude indicators of abundance, and comparative analyses have been used to assess the accuracy of LEK-derived data (De Damasio et al., 2015; Sáenz-Arroyo & Revollo-Fernández, 2016). We compared the catch reduction rate and fitted an exponential decay model (*QtiPlot 0.9.9.7*) as an experimental process to evaluate trends in LEK-derived CPUE and annual landings (Article S1). We then standardized both datasets to z-scores to avoid effects from differences in scales (Fig. S6) and used the Lin Concordance Correlation Coefficient (Lin CCC) to assess agreement between paired values (*DescTools* package; Data and Code) (Lin, 1989; Altman & Altman, 1999) (Article S1; Fig. S6).

### Phase 4: analysis of standardized CPUE data

We performed descriptive statistical analysis and NLR on the standardized database, following the procedures described in “Preliminary Data Evaluation”, to understand long-term abundance trends. We chose NLR for final analyses because (i) analyses conducted in Phases 2 and 3 consistently showed an exponential decay trend, and (ii) standardized CPUE data were not normally distributed (Shapiro–Wilk, *p* = 0.00334), and NLR does not assume normally distributed data (Ritz & Streibig, 2008).

We ran a global sensitivity analysis using Markov-Chain Montecarlo (MCMC) methods in *R 3.4* (*FME* package; Data and Code) to derive the data-dependent probability distribution of the parameters. An MCMC samples from probability distributions to generate an ensemble of parameter values that represent the parameter distribution (Soetaert & Petzoldt, 2010). We ran an MCMC with initial values from the model with best fit (α = 18.911, β = −0.264), with non-informative priors and 5000 iterations. We then estimated the effect of parameter uncertainty on the model output and generated a posterior predictive distribution of the model by taking a sample of the parameter probability function generated by the MCMC, running the model 100 times using a random draw of the parameters in the chain, and adding randomly distributed noise to estimate measurement error (Soetaert & Petzoldt, 2010).

## Results

**Figure 5:**
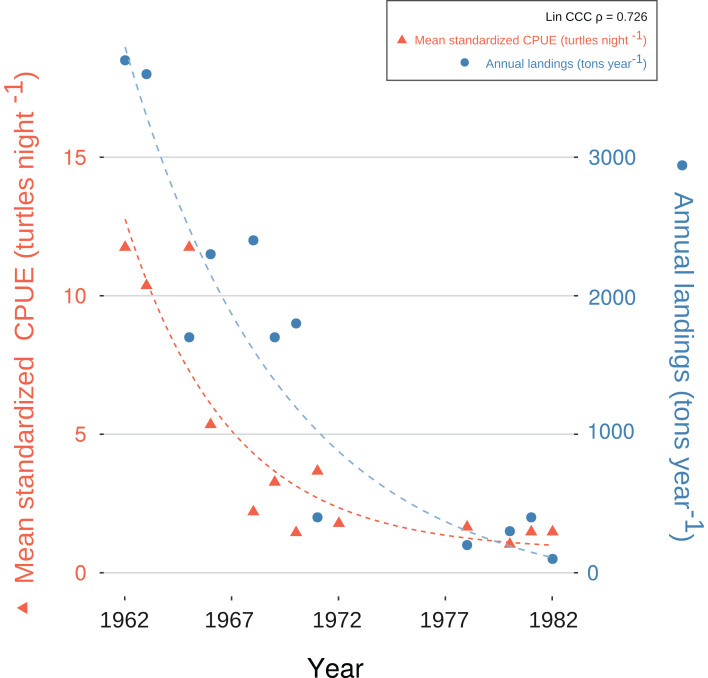
Exponential decay model fitted to standardized catch-per-unit-effort (CPUE) values for *C. mydas* in Bahías de los Ángeles, derived from local ecological knowledge (LEK). Data points are mean, standardized LEK-derived CPUE values for a specific year (red triangles and dotted line; left *Y*-axis) and total annual landings from available fisheries statistics for Bahía de los Ángeles (blue circles and dotted line; right *Y*-axis) (Márquez in Seminoff et al. (2008)). Curves represent suggested trends based on an exponential decay model (details in Article S1). Lin Concordance Correlation Coefficient of paired *z*-scores suggests strong agreement between datasets (see also Fig. S6).

We generated a reliable, standardized green turtle fishery CPUE time-series from 1952 to 1982 by synthesizing and quantifying LEK. Three GLMs fit selection criteria to be used for database standardization, as described in “CPUE Database Standardization” (Table 2). These models showed that year, fishing gear type, vessel capacity, number of nets, net length, and fisher’s experience were significant predictor values for CPUE (Table 5). Given that each of these variables was incorporated into CPUE calculation and standardization, we are confident that both our estimates and standardization procedure were robust. Comparative analysis with fishery landing statistics confirmed accuracy: standardized CPUE and annual landings showed catch declines of 95% and 96%, respectively, and Lin CCC (ρ = 0.726) showed strong agreement (Fig. 5).

**Table 5:** Generalized linear model (GLM) results for the raw catch-per-unit-effort (CPUE) database. The three most parsimonious GLMs for the raw database suggested that fishing gear type, vessel capacity, and number of nets were significant predictor variables for CPUE. Italics indicate significant results at α = 0.95. Asterisks indicate significant result at α = 0.90; this variable was included to ensure robust residuals.

Predictors	Estimate	Std. error	*P*-value
Model 1: log(cpue) ~ Year serial + Experience + Vessel Capacity −1; AIC: 4.422; *D*^2^ = 0.775; df = 32; *e_i_* ~ *N*(0, σ^2^)			
Correlation structure: Auto-regressive, Formula: ∼Year serial			
Year (serialised)	−0.278	0.00434	*0.000*
Experience	0.333	0.0328	*0.000*
Vessel Capacity	0.330	0.0692	*0.000*
Model 2: log(cpue) ~ Year serial + Gear + Total Net Length + Number of Nets + Experience – 1; AIC: 10.215; D^2^ = 0.925; *df* = 20; e_i_ ~ N(0, σ^2^)			
Correlation structure: Auto-regressive, Formula: ∼Year serial			
Year (serialised)	−0.0239	0.0061	*0.0014*
Gear type	0.396	0.0980	*0.0011*
Total net length	−0.150	0.0429	*0.0033*
Number of nets	0.238	0.0750	*0.0062*
Experience	0.0969	0.0494	0.0689*
Model 3: log(cpue) ~ Year serial + Gear + Net Length −1; AIC: −11.710; *D*^2^ = 0.971; df = 32; *e_i_* ~ *N*(0, σ^2^)			
Correlation Structure: Auto-regressive, Formula: ∼Year Serial			
Year (serialised)	−0.0284	0.00465	*0.000*
Gear type	1.324	0.0689	*0.000*
Net length	−1.321	0.0680	*0.000*

**Figure 6:**
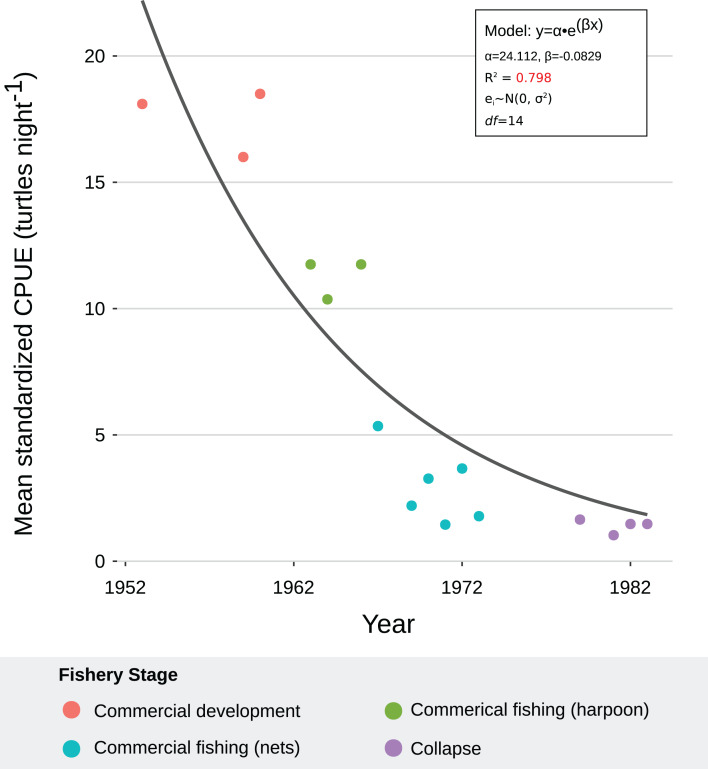
Exponential decay model fitted to mean, standardized catch-per-unit-effort (CPUE) values for *C. mydas* in Bahía de los Ángeles, derived from local ecological knowledge (LEK). Curve represents the nonlinear regression with best fit and robust residuals, based on an exponential decay model. Each data point is a representative, mean, standardized CPUE value for a specific year derived from LEK data. Colors represent fishery stages (see Table 4). Parameter values, standard error, confidence intervals, *t*-values, and *P*-values can be found in Table 6.

All fishers consistently reported a declining trend during the fishery. This was consistent with our models. The NLR with best fit indicated that green turtle abundance declined exponentially through the four phases of the sea turtle fishery, likely driven by large-scale commercial exploitation with increased fishing effort and efficiency from 1960 to 1980 (*R*^2^ = 0.798) (Table 6; Fig. 6). Residual analysis suggested that the model was robust for the data (Table 2). MCMC sensitivity analysis suggested that our model is robust over parameter intervals for α (15–35) and β (−0.12 to −0.06). Parameter values for the model with best fit (α = 24.112, β = −0.0929) occurred within these intervals (Fig. S7). Furthermore, 94% of our data points occurred within the posterior predictive distribution, confirming that the model was a good fit for the data (Fig. S8).

**Table 6:** Results of nonlinear regression with best fit for catch-per-unit-effort estimates derived from local ecological knowledge. Italics indicate significant results at α = 0.95. See also Fig. 6.

Parameter	Estimate	Std. error	95% CI	*t*-value	*P*-value
α	24.112	3.124	[17.413–30.812]	7.719	*2.07e-06*
β	−0.0829	0.0130	[−0.111 to −0.0551]	−6.382	*1.71e-05*

**Note:**

Model: Y ~ α ∙ e^(βx)^; df = 14; *e_i_* ~ *N*(0,σ^2^).

## Discussion

### Integrative methodological innovation

The importance of LEK data is increasingly recognized in conservation science (Lee et al., 2018; Mason et al., 2019). However, there has been reticence in the scientific community regarding the use of LEK due to concerns about accuracy, reliability, and potential biases caused by differences in individual perception, memory, and recollection (O’Donnell, Pajaro & Vincent, 2010; Daw, Robinson & Graham, 2011). We are confident that such issues can be overcome through innovative, transdisciplinary methodologies that incorporate trusted methods from the social sciences and epistemological frameworks for incorporating multiple knowledge systems (Reed, 2008; Tengö et al., 2014; St. John et al., 2014). Our work contributes to overcoming key scientific challenges of using LEK as a consistent source of information by combining rigorous approaches to LEK documentation, synthesis, quantification, and statistical analysis.

We have approached the issues of accuracy, reliability, and recollection bias through several complementary processes. We used ethnography to document LEK, collecting verified, corroborated, and detailed qualitative and numerical data. Ethnographic data allowed for increased accuracy and reliability in comparison with data derived from structured questionnaire-based surveys or interviews alone. This particularly relevant in scenarios of high socio-environmental and biological complexity where multiple variables can affect or bias estimates of species abundance (Crandall et al., 2018; St. John et al., 2014). Ethnographic data also allowed us to understand the trajectory of human impacts on green turtle abundance in detail. This approach allowed us to describe, quantify, and integrate the social, economic, and technological processes that affected the green turtle fishery into our estimates and indices (e.g., changes in fishing gear and displacement capacities, commercial demand, spatial dynamics, etc.). Thus, we incorporated detailed knowledge of the nature and evolution of the green turtle fishery into our models. We reduced cognitive bias and recollection bias by estimating CPUE on the basis of biocultural consensus from multiple, nested knowledge systems rather than direct individual recollection. Finally, we integrated statistical analysis and feedback throughout all phases of our methodology to assure statistical robustness.

The strong concurrence of our LEK-derived CPUE estimates with fishery landing data for the historical fishery years (1962–1982) helps confirm the accuracy of LEK as a source of information for understanding population trends in the recent past (De Damasio et al., 2015; Sáenz-Arroyo & Revollo-Fernández, 2016). Robust model-fitting and sensitivity analyses confirmed statistical reliability. Thus, we are confident that our methods provide practical approaches to the scientific challenges of using LEK in conjunction with ecological modeling through detailed LEK documentation, biocultural consensus, and continuous statistical analysis and feedback.

Spatial dynamics present an interesting area of opportunity for future research building upon our methods. The development of sound approaches to management and conservation requires understanding fishing effort over both space and time (Anticamara et al., 2011; Selgrath et al., 2018). We approached spatial variability through proxies (e.g., propulsion and trip duration, Tables 3 and 4) due to the very high variability and complexity of spatial dynamics over time. Given that our primary focus was on temporal trends in CPUE, these proxies provided a simple index to account for spatial effort (Hilborn & Walters, 1992; Walters, 2003). LEK-based studies of spatial dynamics have primarily been conducted with present-day, data-poor fisheries (cf. Moore et al., 2010; Moreno-Báez et al., 2010; Selgrath et al., 2018). In future, the application of our methods for detailed reconstruction of the spatial dynamics of the green turtle fishery through LEK could have important potential for uncovering historical green turtle spatial distribution and habitat use, and understanding changes in spatial dynamics over broad temporal scales.

We recognize that LEK data is epistemologically distinct from technical data, and have aimed to bridge epistemological gaps and produce a synergistic integration of LEK and scientific methods (cf. Brook & McLachlan, 2005; Tengö et al., 2014). As scientists, we recognize that our research is value-laden and that the inevitable differences between LEK and technical data are more often reflections of epistemological differences or methods of collection than inherent unreliability (Brook & McLachlan, 2005). LEK research requires trust-based collaboration between researchers and communities, a process that can necessitate years of commitment (Brook & McLachlan, 2005). In such contexts, when researchers can elicit and corroborate qualitative data derived from empirically-lived situations (Palmer & Wadley, 2007), synthesize and quantify this data, and submit quantified data to rigorous mathematical analysis, they can assure that LEK-derived estimates are accurate and statistically reliable. Such information is of crucial importance for conservation and management, particularly in scenarios where there is a need for understanding long-term trends; where technical data are scarce or unavailable; or where species are impacted by illegal, unregulated or undocumented exploitation (Duffy et al., 2016; Pauly, 1995; Sáenz-Arroyo & Revollo-Fernández, 2016). Concomitantly, the integration of LEK and scientific knowledge offers the possibilities of incorporating and empowering local conservation processes with peoples previously seen as deleterious agents for those same environments and species of which they hold a vast amount of LEK (cf. Berkes et al., 2005). Lastly, the use of LEK provides comprehensive understanding of complex and dynamic socio-ecological processes while facilitating the creation and implementation of culturally appropriate local solutions to environmental problems (cf. Reed, 2008; Brown, 2010).

### Understanding east pacific green turtle population trends

Our LEK-derived CPUE data provide a baseline abundance of green turtles before large-scale commercial exploitation at a key feeding area in the Gulf of California, and describe population trends prior to ecological monitoring which are essential for establishing conservation and management goals (McClenachan et al., 2016; Seminoff et al., 2003). Our approach provides a historical reference point for the Bahía de los Ángeles foraging population and enables us to better understand contemporary datasets and current population status in the area (Seminoff et al., 2008). Our results suggest that fishery-derived mortality exceeded replacement via reproduction or immigration rates into the feeding areas (Chaloupka & Musick, 1996). Furthermore, although fishing effort and efficiency increased over time, previous CPUE could not be maintained due to the overall decline in green turtle abundance (Hilborn & Walters, 1992). We are confident that CPUE values in the 1950s can be considered an adequate historical baseline abundance level, based on previous research which identified the early 1960s as a period when human impacts precipitated a major decline in green turtle abundance in the Gulf of California (Early-Capistrán et al., 2018).

Future research that pairs LEK-derived estimates with contemporary in-water monitoring and nesting data can provide fundamental insights for conservation status evaluations such as those conducted under the auspices of the IUCN Red List (Mazaris et al., 2017; Seminoff & Shanker, 2008). Such long-term perspectives are generally not attainable via scientific monitoring efforts alone, especially considering that although sea turtles have been exploited worldwide for centuries or millennia, even the longest tenured sea turtle monitoring programs only started the 1960s (Balazs & Chaloupka, 2004; Bjorndal, Bolten & Chaloupka, 2005; Chaloupka & Limpus, 2001; Márquez, 1996).

In the case of BLA, existing baseline data from 1995 correspond to a decimated population, and would thus be prone to over-estimating the degree of initial recovery observed from the early 2000s onward (Delgado-Trejo, 2016; Pauly, 1995; Seminoff et al., 2015). Currently, scientific surveys are conducted monthly using CPUE as an index. Catch effort is variable within specific parameters, using 100–200 m set-nets and 12–24 h soak times (Koch, 2013; Seminoff et al., 2008). In future, our standardized LEK-derived CPUE estimates can be integrated with standardized monitoring data to provide a long-term view of green turtle abundance at this index feeding area. Integration of past trends with modern-day survey data is crucial for evaluating the overall conservation status of the East Pacific green turtle with references to baseline abundance levels prior to large-scale commercial exploitation (Broderick et al., 2006; Seminoff & Shanker, 2008; Wildermann et al., 2018).

## Conclusions

Our reconstruction of baseline conditions revealed an exponential decline in green turtle abundance between 1960 and 1980 at Bahía de los Ángeles, one of the most important and productive green turtle commercial fishing areas in the eastern Pacific Ocean (Caldwell, 1963; Early-Capistrán et al., 2018). As scientific monitoring began only in 1995 after population collapse, no pre-exploitation baseline data were available to evaluate current abundance and conservation status (Seminoff et al., 2008). Our LEK-derived data can now provide historical context and a reliable baseline abundance estimate for this green turtle population. We are confident that future studies integrating our LEK-derived estimates with current scientific monitoring data from both foraging habitats and nesting beaches will yield a holistic, long-term perspective of green turtle abundance, conservation, and population dynamics in the eastern Pacific.

Beyond reconstructing green turtle abundance, our methodology may be exported to parallel cases dealing with the conservation and monitoring of other long-lived species that are fished as it can unravel complex phenomena by combining LEK and ecological modeling. We provide a framework to overcome the challenges of documenting and quantifying LEK, and bridge practical and epistemological gaps (Mistry & Berardi, 2016; Mukherjee et al., 2018). This approach provides a way to deal with variation in individual memory using corroborated data and collectively produced knowledge, to simplify and manage large volumes of qualitative information, and to translate qualitative data into a format compatible with ecological modeling (Bélisle et al., 2018).

We recognize that of LEK and derived population abundance estimates are technically and epistemologically distinct from data obtained under experimental conditions. Nevertheless, they can provide a robust description of significant inflection points in abundance trends that would be less-resolved if analyses were limited to scantly-available technical data (Pauly, 1995; Sáenz-Arroyo & Revollo-Fernández, 2016). LEK-based and integrative approaches can provide long-term information where scientific monitoring data are scarce or unavailable, and contribute to collaborative knowledge production (Barrios-Garrido et al., 2018; Lee et al., 2018; Mistry & Berardi, 2016). While our methods are most readily adapted to marine fauna such as marine mammals, reptiles, teleost fish, and long-lived invertebrates, this approach can also be modified and applied to terrestrial and freshwater biota. We trust that future research that rigorously integrates social and ecological science can help address challenges for conservation and management in the context of global change and biodiversity loss (Mukherjee et al., 2018; Sutherland et al., 2018).

## Supplemental Information

10.7717/peerj.9494/supp-1 Supplemental Information 1 Supplemental Information for Methods and Results.

10.7717/peerj.9494/supp-2 Supplemental Information 2 Ethical Guidelines and Protocols.

10.7717/peerj.9494/supp-3 Supplemental Information 3 Qualitative-Quantitative Data Analysis.

10.7717/peerj.9494/supp-4 Supplemental Information 4 Number and type of interviews.

10.7717/peerj.9494/supp-5 Supplemental Information 5 Fieldwork inventory.

10.7717/peerj.9494/supp-6 Supplemental Information 6 Text box with example extract of a field journal entry (translated from Spanish and abbreviated). Bold type indicates numerical data. Date and study site are indicated at the beginning of the journal entry. Content is grouped in blocks of time. The approximate time of day and location, along with a general description of the activity and a cryptic indicator of the collaborator(s), are included in the heading of each block of time. Italics indicate categories adapted from the Outline of Cultural Materials (Murdock et al., 2008) at the beginning of each paragraph. Analysis, commentary, and cross-references are separated from observations with footnotes ([1]) at the end of each paragraph. Specific topics of interest are indexed using hashtags (#). […] indicates redacted segments.

10.7717/peerj.9494/supp-7 Supplemental Information 7 Text box with example of a fisher summary file (translated from Spanish). The header includes the cryptic indicator for the collaborator and a list of their corresponding field journal entries and interview transcripts. Content includes summarized qualitative and quantitative information, quotes, follow-up notes, and commentary.

10.7717/peerj.9494/supp-8 Supplemental Information 8 Linear regression of maximum catch-per-unit-effort data for Bahía de los Ángeles compiled in 2012-2013 (adapted from Early-Capistrán, 2014).

10.7717/peerj.9494/supp-9 Supplemental Information 9 Example of quantitative textual analysis. Cultural material codes used most frequently in field journals. ****Blue text indicates customized codes adapted from Murdock et al. (2008).

10.7717/peerj.9494/supp-10 Supplemental Information 10 Example of quantitative textual analysis. Wordcloud of words and phrases used most frequently in field journals (translated from Spanish). Size indicates relative frequency.

10.7717/peerj.9494/supp-11 Supplemental Information 11 Raw and standardized LEK-derived catch-per-unit-effort (CPUE) values for for *C. mydas* in Bahía de los Ángeles. Data points represent raw CPUE (red crosses) and standardized CPUE (blue circles). Data points for years with multiple CPUE values are included.

10.7717/peerj.9494/supp-12 Supplemental Information 12 Standardized, LEK-derived catch-per-unit-effort (CPUE) values for *C. mydas* in Bahía de los Ángeles. Blue circles represent all standardized CPUE values, including years with multiple data points. Red crosses represent the mean standardized CPUE values for a given year. Mean, standardized CPUE values (red crosses) were used for final analyses.

10.7717/peerj.9494/supp-13 Supplemental Information 13 Comparison of z-scores for catch-per-unit-effort (CPUE) derived from local ecological knowledge (LEK) and annual landings for *C. mydas* in Bahía de los Ángeles. Z-scores for LEK-derived CPUE (blue circles) and annual landings from fisheries statistics available for 1962-1982 for Bahía de los Ángeles (red circles) (Márquez in Seminoff et al., 2008)

10.7717/peerj.9494/supp-14 Supplemental Information 14 Pairs plot of MCMC sensitivity analysis. Pairs plot of the MCMC sensitivity analysis of for both parameters of the nonlinear regression model with best fit (eqn. 2, Figure 6 in main text). The upper panel shows the pairwise relationship of both parameters, the lower panel shows correlation coefficients, and the diagonal shows marginal distribution for each parameter represented by a histogram.

10.7717/peerj.9494/supp-15 Supplemental Information 15 Posterior predictive distribution of the nonlinear regression model with best fit. Posterior predictive distribution of the nonlinear regressios model with best fit (eqn. 2, Figure 6 in main text) obtained from a randomly selected subset of 100 parameter combinations from the MCMC chain and randomly distributed noise to estimate measurement error. Black dots represent mean standardized catch-per-unit-effort values for *C. mydas* en Bahía de los Ángeles.

10.7717/peerj.9494/supp-16 Supplemental Information 16 Quantitative datasets and R code. See README.txt for further information

10.7717/peerj.9494/supp-17 Supplemental Information 17 COREQ Checklist.

## Additional Information and Declarations

### Data Availability

The following information was supplied regarding data availability: Quantitative datasets without personally identifiable information and R code are available as a Supplemental File and and GitHub (https://github.com/earlycapistran/PeerJ). Due to the sensitive and confidential nature of ethnographic data, all primary ethnographic data (including field notes, field journals, photographs, archival materials, audio and video recordings, and transcriptions) are stored in secure archives and may only be accessed by the core research team (Michelle-María Early-Capistrán, Elena Solana-Arellano, and F. Alberto Abreu-Grobois), in compliance with the ethical guidelines of the International Society of Ethnobiology.
