# Supplementary material for: Quantifying local ecological knowledge to model historical abundance of long-lived, heavily-exploited fauna
Source: PeerJ. 2020 Jul 20;8:e9494. doi: 10.7717/peerj.9494 (PMC7377249; doi:10.7717/peerj.9494)
Supplement: Supplemental Information 1 [file peerj-08-9494-s001.pdf]

SUPPORTING INFORMATION

ARTICLE S1

Main text: Quantifying Local Ecological Knowledge to Model Historical Abundance of Long-lived, Heavily-Exploited Fauna

Journal: PeerJ

Authors: Early-Capistrán, Michelle-María[1], Solana-Arellano, Elena[2], Abreu-Grobois, Federico-Alberto[3], Narchi, Nemer E.[4], Garibay-Melo, Gerardo[5], Seminoff, Jeffrey A.[6], Koch, Volker[7], Sáenz-Arroyo, Andrea[8]

[1] Posgrado en Ciencias del Mar y Limnología, Universidad Nacional Autónoma de México, Mexico City, Mexico

[2] Departamento de Ecología Marina, Centro de Investigación Científica y Educación Superior de Ensenada, Ensenada, Baja California, Mexico.

[3] Instituto de Ciencias del Mar y Limnología – Unidad Académica Mazatlán, Universidad Nacional Autónoma de México, Mazatlán, Sinaloa, Mexico.

[4] CoLaboratorio de Oceanografía Social/Centro de Estudios de Geografía Humana, El Colegio de Michoacán - Sede La Piedad, La Piedad, Michoacán, Mexico

[5] Posgrado en Manejo de Ecosistemas de Zonas Áridas. Universidad Autónoma de Baja California, Ensenada, B.C., Mexico.

[6] NOAA – Southwest Fisheries Science Center, La Jolla, California, U.S.A.

[7] Deutsche Gesellschaft für Internationale Zusammenarbeit (GIZ) GmbH, Bonn Germany

[8] Departamento de Conservación de la Biodiversidad. El Colegio de la Frontera Sur (ECOSUR), San Cristóbal de Las Casas, Chiapas, Mexico

EPISTEMOLOGY

We engaged with epistemology, ontology, phenomenology, hermeneutics, and heuristics to inform which methodologies were best suited and how they could be integrated (Moon et al., 2019). We integrated multiple ways of knowing through a common epistemology developed *ad hoc* through an active dialogue between knowledge systems and disciplinary approaches (Miller et al., 2008; Tengö et al., 2014). We took a phenomenological approach to the research topic, seeking to understand commonly observed phenomena (e.g.: green turtle populations observed by commercial fishers), rather than individual experience (e.g.: memories recalled by an individual fisher) (Converse, 2012).

HISTORIOGRAPHICAL RESEARCH

Historiographical research involves reviewing primary sources such as government records or historical texts, which are read critically and situated within historical processes (Brettell, 1998; Bernard, 2011; Early-Capistrán et al., 2018). We analyzed documents' internal and external validity based on hermeneutic and semiotic analysis (Denzin & Lincoln, 1994), with sensitivity to the social, political, and historical context in which they were generated and considering the impact of cultural contact, conquest, and colonialism as historical processes that can bias texts (Brettell, 1998). We identified sources of bias (observer bias, informer bias, and authorial ethnocentrism) by systematically analyzing who collected the data; how, why, under what conditions the information was produced or

collected; and towards whom the texts were directed (Brettell, 1998; Bernard, 2011; Early-Capistrán et al., 2018).

ETHNOGRAPHY

Qualitative and Quantitative Data and analyses

Ethnography allows for the capture of both qualitative and quantitative data, as well as for both quantitative and qualitative analyses of data. We used both types of data and analytical approaches throughout (Table S1).

Integrating data from 2012-2013

We integrated data and materials from previous ethnographic research on the green turtle fishery in BLA in 2012-2013 (Tables S2, S3) that included participant observation, semi-structured, in-depth, and informal interviews with sea turtle fishers (n=16) and community members at large (Early Capistrán, 2014; Early-Capistrán et al., 2018). It should be noted that, unfortunately, 29% of sea turtle fishers passed away between 2012 and 2017, making this previous information particularly valuable for current research. We integrated data from past field seasons —including interviews, transcriptions, and audio and video recordings (Table S3) — by applying the same processing and synthesis as data compiled in 2017-2018. This process increased the effective sample size of sea turtle fishers (n=22) and substantially increased available materials and information.

Field Journal Entries

Field notes were captured continually throughout the day, and logged by blocks of time for later entry into the field journal in digital format (.txt) (Table S4). One field journal entry was captured daily. Each entry included all notes taken as well as additional information observed throughout the day. Field journal entries were detailed and extensive, ranging from 2,000-10,000 words or more. Field journal writing is a key part of ethnographic research involving two to eight hours per working day, with a working day in the field lasting 12-16 hours on average (Bernard, 2011). Field journal entries were indexed and coded for future reference and classification.

The date and study site were indicated at the beginning of the journal entry. Content is grouped in blocks of time. The approximate time of day and location, along with a general description of the activity and a cryptic indicator of the collaborator(s), were included in the heading of each block of time. Categories from the Outline of Cultural Materials (Murdock et al., 2008), a series of standardized numerical cades used to organize ethnographic data, were included at the beginning of each paragraph. We added customized codes for this research (Bernard, 2011) (e.g.: 226.5.1, Historical sea turtle fishing; 441.1, sea turtle commerce; 226.8, fishing grounds). Analysis, commentary, and cross-references were separated from observations with footnotes ([1]) at the end of each paragraph. Analyses were cross-referenced between journal entries when applicable. Specific topics of interest were indexed using hashtags (#). See Table S4 for an abbreviated example of a field journal entry.

Numerical data capture

Numerical data was captured from field journals and interview transcriptions (see bold type in Table S4). Note that we do not refer to these values as quantitative data. These initial values were corroborated, verified, and converted into reliable, quantitative data through the processes described in Phases 2-4 of the Methods section in the main text.

We compiled summary files for each fisher, with synthesized biographical, qualitative, and numerical data from field journals, notes, and interviews, as well as references to each interview, note, and journal entry associated with the fisher (Table S5). This allowed us to quickly access and cross-reference information. Each numerical data point used in calculations and modeling processes was linked to a summary file, and outlying data could be contextualized, evaluated, and cross-referenced.

ETHICS

Fieldwork was conducted in accordance with nationally and internationally recognized ethical guidelines for ethnobiological and ethnographic research, as established in the Code of Ethics of the International Society of Ethnobiology (ISE) and the Latin American Society of Ethnobiology (SOLAE) (International Society of Ethnobiology, 2006; [SOLAE] Sociedad Latinoamericana de Etnobiología, 2014), and approved by the Bioethics Committee of the Centro de Investigación Científica y de Educación Superior de Ensenada (Approval Number 2S.3.1). We obtained permission from local authorities, and clearly disclosed the aims and objectives of the research, institutional backing and funding sources to both local authorities and participants, and provided the research team's contact

information (International Society of Ethnobiology, 2006). All participation was voluntary, and we clearly communicated to participants the right to reserve responses to any questions or to cease participating in the research at any time (International Society of Ethnobiology, 2006; Bernard, 2011). We gained informed, verbal consent to carry out or record interviews, and to take photographs or produce audio or video recordings (International Society of Ethnobiology, 2006). We chose oral consent was chosen as it was not deemed culturally appropriate to ask participants to sign a consent document, and because some participants were not comfortable with written language (Wedemeyer-Strombel et al., 2019).

We ensured anonymity for participants, and also gave the option to be identified and/or credited (International Society of Ethnobiology, 2006; [AAA] American Anthropological Association, 2012). Field notes and journals are stored privately in encrypted form, with names replaced by cryptic indicators, to assure confidentiality (International Society of Ethnobiology, 2006; Bernard, 2011; [AAA] American Anthropological Association, 2012). We offered contributors copies of interview recordings, and committed to provide copies of research to the community, along with culturally appropriate materials for communicating results, such as videos or posters in Spanish. We also asked for permission to use images or compiled materials, and committed to recognizing the authorship of said materials (International Society of Ethnobiology, 2006).

As researchers, we have a responsibility to be aware of and comply with systems for management of knowledge, particularly in regard to sensitive issues; to guarantee confidentiality; and to protect the rights local contributors' privacy and anonymity at their

discretion (International Society of Ethnobiology, 2006). Due to the sensitive and confidential nature of ethnographic data, all primary ethnographic data (including, but not limited to, field notes, field journals, photographs, archival materials, audio and video recordings, and transcriptions) are held in the custody and possession of the first author M.M.E.C., and stored and preserved indefinitely in secure archives. Ethnographic data may only be accessed by the core research group (M.M.E.C., F.A.A.G, and E.S.A.), in compliance with the ethical guidelines of the International Society of Ethnobiology (International Society of Ethnobiology, 2006; Pels et al., 2018).

CPUE CALCULATION

Individual memory and knowledge varied among contributors, and all fishers reported substantial variability in captures throughout the fishery. Furthermore, some fishers reported average catches (a catch in a night that was neither good nor bad) or mode catches (number of turtles caught most frequently in a single night), while others reported intervals of CPUE values or trip times to fill a vessel to capacity. Thus, we developed the framework described in Figure 3 of the main text to make systematic inferences to (i) standardize response terms and (ii) deal with data gaps and calculate CPUE by complementing information from less experienced fishers with that of experts. Specific procedures are described in detail in the following sub-sections.

7Commercial dynamics

The green turtle fishery followed the same catch dynamic throughout all stages: fishers would make trips of varying duration until they filled their vessel or exhausted food and water supplies, and then returned to shore to deliver the catch. Turtle fishing was carried out at night. Catches from multiple crews were gathered in pens by merchants, until a sufficient number was caught to load onto 3-4 ton trucks for transport to the market city of Ensenada, near the U.S. border, ~700km northwest (Early-Capistrán et al., 2018). Fishing activity was generally higher in summer (Caldwell, 1963). During some stages, crews would off-load to boats (~7 tons) from canoes or skiffs to allow for profitable trips to more distant fishing grounds. In such cases, we verified with fishers that their reported CPUE referred specifically to their crew and vessel (canoe or skiff with corresponding gross tonnage), separately from the total for the boat.

Calculating fishing time

We obtained minimum (1 night) and maximum (10 nights) trip duration limits from interviews. Trip duration had a maximum limit because fishers carried all supplies (food, water, fishing gear, etc.) with them. Water supply was an important limiting factor in the desert environment. During semi-structured and in-depth interviews, we asked about average trip duration, spatial distribution of fishing, and travel time to the fishing grounds used most frequently during each of the fishery stages.

Fishers reported trip time (total time from leaving port until returning with a vessel at full capacity) in number of days, which we converted to total hours. We calculated

fishing time using a modified application of the formula proposed by Hilborn and Walters (1992) by first calculating the number of days spent at fishing grounds:

$$T_{grounds} = (T_{total} - T_{displacement})/24 \qquad \text{(eqn. S1)}$$

Where $T_{grounds}$ (days) is time spent at fishing grounds; T_{total} is trip time (hours), total time from leaving port until returning with a vessel at full capacity; $T_{displacement}$ is travel time between port and fishing grounds (hours), calculated as mean displacement time to the fishing grounds used most frequently during the fishery stage.

We converted $T_{grounds}$ to hours and calculated active fishing time:

$$T_{fishing} = (T_{grounds} - T_{activities}) / 12 \qquad \text{(eqn. S2)}$$

Where $T_{fishing}$ is active fishing time (hours) and $T_{activities}$ (hours) are non-fishing activities (catch processing, sleep, meals, repairs, etc.).

We binned time budgets into two blocks of 12 hours, based on the assumption that active fishing was carried out in ~12 hour blocks, with remaining time budgets allocated to non-fishing activities in ~12 hour blocks. We based this assumption on fishers' consistent reports that fishing activity was generally carried out from dusk until dawn, regardless of gear type. However, we must point out that for any given day, fishing durations with either gear type were variable. Harpooners would work until reaching vessel capacity and were limited by weather conditions, tides, and propulsion, among other variables, meaning that active fishing time could sometimes be less than 12 hours (e.g., on a good night when vessels were filled quickly, when changing weather did not allow for continuous fishing, etc.). In the case of nets, set times could be greater than 12 hours if turtles were scarce or if

weather did not allow fishers to remove nets at customary 12 hour intervals. Given the general trends and the nature of the data, we consider our 12 hour blocks to be reflective of the vast majority of fishing effort.

Calculating dates for CPUE

If fishers did not recall specific dates, we used salient events in their personal life (e.g.: marriage, birth of a child, etc.) or events in the green turtle fishery (the introduction of nets, the introduction of motors, restrictions to fisheries cooperatives or temporary bans, the total ban on turtle captures, etc.) as prompts to situate responses in a time frame within fisheries stages. If, in response to prompts, fishers provided time frames or intervals rather than specific dates, we assigned dates based on end-points within the fishery stage. For example, if they referred to early or late years within a stage, we used the first year or last year respectively (e.g.: "during my first years fishing with set-nets", "during my last years as a harpooner", etc.). If they referred to the stage in general terms we used the median year (e.g.: "when I was a harpooner"). If a fisher began or ended their career during a specific stage, the respective career dates and stage dates were used as end-points. For example, if a fisher worked from the beginning of the "Overfishing (net)" stage (1966-1972) until 1968, 1966 and 1968 were used as end-points.

Calculating number of turtles caught

Most fishers reported catches in number of turtles, but some reported total weight (kg). In these cases, we calculated the number of turtles caught by dividing gross vessel tonnage by

mode turtle mass (kg). Vessel types and capacity were documented through ethnographic research, and binned as an ordinal variable (see main text, Table 3). In cases where fisher off-loaded to boats, we verified CPUE values specifically for their crews' canoe or skiff.

Fishers reported turtle sizes in kilograms, as they were paid by weight. Green turtle size distribution was highly variable and likely declined with fishing effort, as captures of large (>150kg) turtles became less frequent in later years. However, we assumed that mode green turtle size was constant across fishery stages. We based this assumption on fishers' reports of consistent mode sizes across time (Early-Capistrán et al., 2018). This is consistent with mixed juvenile/adult foraging groups with a slight juvenile bias —such as BLA, where ~56% of individuals are juveniles—found in green turtle foraging habitats worldwide (Seminoff et al., 2003, 2015). We based our value for mode size on scientific monitoring data, corroborated with the mode weight (50kg) reported by fishers as far back as 1940, which is congruent with scientific monitoring data from 2001-2013 (Mo=45.0) (Grupo Tortuguero de las Californias A.C., 2008; Área Natural Protegida de Flora y Fauna Islas del Golfo de California & Comisión Nacional de Áreas Naturales Protegidas, 2013).

Adjusting for seasonality

Expert turtle fishers noted that spatial distribution of fishing and capture rates varied greatly by season due to winter dormancy behavior. In winter, turtles were less mobile and less susceptible to gear. Fishers had to travel farther to fill their quotas, and generally had smaller catches. We then standardized questions to include references to seasonality, and to focus reports of catches primarily on summer months, when fishing was most active, to

reduce the bias generated by changes in fishing dynamics during winter and to account for differences in seasonality.

Spatial distribution of fishing

Spatial distribution of fishing was highly variable throughout the chronology, due to the search for hot-spots and aggregations and due to the effects of seasonality and changes in abundance. However, fishers generally covered greater distances as green turtle aggregations close to port became less frequent year-round, and as outboard motors facilitated trips of greater distance in shorter times. Thus, we used propulsion methods and trip times as proxies for spatial distribution.

RESIDUAL ANALYSIS

We incorporated residual analyses throughout model fitting processes to ensure that model assumptions were met, and that to evaluate goodness of fit and model robustness. We ensured that residuals for all models reported in the results were normally distributed, randomly distributed, independent, homoscedastic, and had zero mean ($e_i \sim N(0, \sigma^2)$). Tests and criteria used to evaluate residual auto-correlation are reported in Table 2 in the main text.

Evaluating residual auto-correlation in NLR

We integrated a novel procedure to assess residual auto-correlation in NLR models. Currently, recommendations for evaluating residual auto-correlation in nonlinear regression are limited to visual analysis of the residual lag plot and runs test for randomness (e.g., Ritz & Streibig, 2008; Baty et al., 2015). Along with visual evaluation, we incorporated a formal statistical test by running a Pearson correlation test of the raw residuals vs. lagged residuals (H_o: $\rho = 0$, H_a: $\rho \neq 0$). The Pearson correlation test suggested that there was not significant correlation between raw and lagged residuals (r=0.434, p=0.108). Additionally, we fitted a linear model to the raw residuals based on lagged residuals. Results of the regression were not significant (p=0.108, R^2=0.124), further confirming the lack of residual auto-correlation.

COMPARATIVE ANALYSIS OF CPUE-TOTAL LANDINGS

We used QtiPlot 0.9.9.7 to fit an exponential decay model (eqn. S3) separately to both standardized, LEK-derived CPUE and fisheries statistics for BLA (annual landings in tons, 1962-1982) (Márquez cited in Seminoff et al., 2008) for an experimental evaluation of general trends in both data sets:

$$\beta_0+\beta_1 * \exp^{(-x/t)} \qquad \text{(eqn. S3)}$$

Where β_0 is an offset value, β_1 is amplitude, and t is e-folding time. It must be noted that our objective was not provide a single model that best described both sets, but to evaluate if

both could be described mathematically in broadly similar terms, and ascertain if we could proceed to test agreement between the two datasets.

This experimental process yielded high R^2 values for both LEK-derived CPUE (R^2=0.848) and fisheries statistics (R^2=0.845), although residuals for LEK-derived CPUE were non-normal at 5% significance (Shapiro-Wilk p=0.02). We did not report detailed results for this model-fitting process in the main text, as it was not the best fit for LEK-derived data. However, this process suggested that both datasets share a similar tendency and can be broadly described in similar terms. Based on this analysis, we considered that the two datasets have a shared tendency, and chose the Lin CCC to further analyze and evaluate agreement evaluate agreement (Lin, 1989).

REFERENCES

[AAA] American Anthropological Association. 2012. *2012 Ethics Statement*.

Área Natural Protegida de Flora y Fauna Islas del Golfo de California, Comisión Nacional de Áreas Naturales Protegidas. 2013. *Monitoreo de tortugas marinas, 2009-2013*. Bahía de los Ángeles, B.C.: Comisión Nacional de Áreas Naturales Protegidas.

Baty F, Ritz C, Charles S, Brutsche M, Flandrois J-P, Delignette-Muller M-L. 2015. A Toolbox for Nonlinear Regression in *R*: The Package **nlstools**. *Journal of Statistical Software* 66. DOI: 10.18637/jss.v066.i05.

Bernard HR. 2011. *Research Methods in Anthropology: Qualitative and Quantitative Approaches*. New York: AltaMira Press.

Brettell CB. 1998. Fieldwork in the Archives: Methods and Sources in Historical Anthropology. In: Bernard HR ed. *Handbook of Methods in Cultural Anthropology*. Walnut Creek, California: AltaMira Press, 513–546.

Caldwell DK. 1963. The sea turtle fishery of Baja California, Mexico. *California Fish and Game* 49:140–151.

Converse M. 2012. Philosophy of phenomenology: how understanding aids research. *Nurse Researcher* 20:28–32. DOI: 10.7748/nr2012.09.20.1.28.c9305.

Denzin NK, Lincoln YS (eds.). 1994. *Handbook of Qualitative Research*. London: Sage Publications.

Early Capistrán MM. 2014. Análisis diacrónico de la explotación, abundancia y talla de Chelonia mydas en la península de Baja California, 12,000 A.P.-2012. Maestría en Ciencias Thesis. México D.F.: Universidad Nacional Autónoma de México.

Early-Capistrán M-M, Sáenz-Arroyo A, Cardoso-Mohedano J-G, Garibay-Melo G, Peckham SH, Koch V. 2018. Reconstructing 290 years of a data-poor fishery through ethnographic and archival research: The East Pacific green turtle (Chelonia mydas) in Baja California, Mexico. *Fish and Fisheries* 19:57–77. DOI: 10.1111/faf.12236.

Grupo Tortuguero de las Californias A.C. 2008. *Datos de monitoreo de tortugas 2001-2008*. La Paz, B.C.S.: Grupo Tortuguero de las Californias A.C.

Hilborn R, Walters CJ. 1992. *Quantitative Fisheries Stock Assessment: Choice, Dynamics and Uncertainty*. Dordrecht: Springer Science+Business Media.

International Society of Ethnobiology. 2006. ISE Code of Ethics (with 2008 additions).

Lin LI-K. 1989. A Concordance Correlation Coefficient to Evaluate Reproducibility. *Biometrics* 45:255. DOI: 10.2307/2532051.

Miller TR, Baird TD, Littlefield CM, Kofinas G, Chapin III FS, Redman CL. 2008. Epistemological Pluralism: Reorganizing Interdisciplinary Research. *Ecology and Society* 13:art46. DOI: 10.5751/ES-02671-130246.

Moon K, Blackman DA, Adams VM, Colvin RM, Davila F, Evans MC, Januchowski-Hartley SR, Bennett NJ, Dickinson H, Sandbrook C, Sherren K, St. John FAV, van Kerkhoff L, Wyborn C. 2019. Expanding the role of social science in conservation through an engagement with philosophy, methodology, and methods. *Methods in Ecology and Evolution*. DOI: 10.1111/2041-210X.13126.

Murdock GP, Clellan SF, Hudson AE, Kennedy R, Simmons LW, Whiting JWM. 2008. *Outline of Cultural Materials. 6th revised edition with Modifications*. New Haven: Human Relations Area Files.

Pels P, Boog I, Henrike Florusbosch J, Kripe Z, Minter T, Postma M, Sleeboom-Faulkner M, Simpson B, Dilger H, Schönhuth M, von Poser A, Castillo RCA, Lederman R, Richards-Rissetto H. 2018. Data management in anthropology: the next phase in ethics governance? *Social Anthropology* 26:391–413. DOI: 10.1111/1469-8676.12526.

Ritz C, Streibig JC. 2008. *Nonlinear regression with R*. New York: Springer.

Seminoff JA, Allen CD, Balazs G, Dutton PH, Eguchi T, Haas HL, Hargrove S, Jensen M, Klemm DL, Lauritsen AM, MacPherson SL, Opay P, Possardt EE, Pultz S, Seney E, Van Houtan KS, Waples RS. 2015. *Status Review of the Green Turtle (Chelonia mydas) Under the Endangered Species Act*. San Diego, USA: NOAA.

Seminoff JA, Jones TT, Resendiz A, Nichols WJ, Chaloupka MY. 2003. Monitoring green turtles (Chelonia mydas) at a coastal foraging area in Baja California, Mexico: multiple indices to describe population status. *Journal of the Marine Biological Association of the UK* 83:1355–1362.

Seminoff JA, Reséndiz-Hidalgo A, Jiménez de Reséndiz B, Nichols WJ, Todd-Jones T. 2008. Tortugas marinas. In: Danemann G, Ezcurra E eds. *Bahía de los Ángeles: recursos naturales y comunidad : línea base 2007*. Tlalpan, México D.F.; San Diego, Calif.: Secretaría de Medio Ambiente y Recursos Naturales ; San Diego Natural History Museum, 457–494.

[SOLAE] Sociedad Latinoamericana de Etnobiología. 2014. Código de ética para la investigación, la investigación-acción y la colaboración etnocientífica en América Latina. *Etnobiología* 12:36.

Tengö M, Brondizio ES, Elmqvist T, Malmer P, Spierenburg M. 2014. Connecting Diverse Knowledge Systems for Enhanced Ecosystem Governance: The Multiple Evidence Base Approach. *AMBIO* 43:579–591. DOI: 10.1007/s13280-014-0501-3.

Wedemeyer-Strombel KR, Peterson MJ, Sanchez RN, Chavarría S, Valle M, Altamirano E, Gadea V, Sowards SK, Tweedie CE, Liles MJ. 2019. Engaging Fishers' Ecological Knowledge for Endangered Species Conservation: Four Advantages to Emphasizing Voice in Participatory Action Research. *Frontiers in Communication* 4:30. DOI: 10.3389/fcomm.2019.00030.
