# Supplementary material for: Quantifying local ecological knowledge to model historical abundance of long-lived, heavily-exploited fauna
Source: PeerJ. 2020 Jul 20;8:e9494. doi: 10.7717/peerj.9494 (PMC7377249; doi:10.7717/peerj.9494)
Supplement: Supplemental Information 2 [file peerj-08-9494-s002.pdf]

SUPPORTING INFORMATION

ARTICLE S2

ETHICAL GUIDELINES AND PROTOCOLS

Main text: Quantifying Local Ecological Knowledge to Model Historical Abundance of Long-lived, Heavily-Exploited Fauna

Journal: PeerJ

Authors: Early-Capistrán, Michelle-María[1], Solana-Arellano, Elena[2], Abreu-Grobois, Federico-Alberto[3], Narchi, Nemer E.[4], Garibay-Melo, Gerardo[5], Seminoff, Jeffrey A.[6], Koch, Volker[7], Sáenz-Arroyo, Andrea[8]

1 Posgrado en Ciencias del Mar y Limnología, Universidad Nacional Autónoma de México, Mexico City, Mexico
2 Departamento de Ecología Marina, Centro de Investigación Científica y Educación Superior de Ensenada, Ensenada, Baja California, Mexico.
3 Instituto de Ciencias del Mar y Limnología – Unidad Académica Mazatlán, Universidad Nacional Autónoma de México, Mazatlán, Sinaloa, Mexico.
4 CoLaboratorio de Oceanografía Social/Centro de Estudios de Geografía Humana, El Colegio de Michoacán - Sede La Piedad, La Piedad, Michoacán, Mexico
5 Posgrado en Manejo de Ecosistemas de Zonas Áridas. Universidad Autónoma de Baja California, Ensenada, B.C., Mexico.
6 NOAA – Southwest Fisheries Science Center, La Jolla, California, U.S.A.
7 Deutsche Gesellschaft für Internationale Zusammenarbeit (GIZ) GmbH, Bonn Germany
8 Departamento de Conservación de la Biodiversidad. El Colegio de la Frontera Sur (ECOSUR), San Cristóbal de Las Casas, Chiapas, Mexico

ETHICAL GUIDELINES

Fieldwork was conducted in accordance with nationally and internationally recognized ethical guidelines for ethnobiological and ethnographic research, as established in the Code of Ethics of the International Society of Ethnobiology (ISE) and the Latin American Society of Ethnobiology (SOLAE) (International Society of Ethnobiology, 2006; Sociedad Latinoamericana de Etnobiología, 2014).

The research team obtained permission from local authorities after providing all relevant information regarding the research (objectives, aims, funding sources, possible risks and benefits, and contact information). All relevant information was also provided to participants, as is described in detail in the following sections (International Society of Ethnobiology, 2006).

All participation in the study was voluntary, and all participants were given the option to reserve the answers to questions, refuse to participate, or stop participating at any moment without providing reasons or motives, and with no penalization (International Society of Ethnobiology, 2006). Oral informed consent was obtained from all participants prior to the start of interviews. All participants were also asked if the consented to being recorded in audio and/or video, and/or photographed in addition to the interview (International Society of Ethnobiology, 2006). Oral consent was chosen as it was not deemed culturally appropriate to ask participants to sign a consent document, and because some participants were not comfortable with written language (International Society of Ethnobiology, 2006; Wedemeyer-Strombel et al., 2019). When possible, oral consent was recorded in audio or video. Information required for prior informed consent was provided

by the research team, following a flexible script which included the aims; purpose; funding bodies; possible risks and benefits; confidentiality; and voluntary participation (Article S3).

The research team recognizes its responsibility to be aware of and comply with systems for management of knowledge, particularly in regard to sensitive issues; to guarantee confidentiality; and to protect the rights local contributors' privacy and anonymity at their discretion (International Society of Ethnobiology, 2006). The contributors' participation, and the information derived from it, is confidential. Field notes and journals are stored privately in encrypted form, with names replaced by cryptic indicators, to assure confidentiality (International Society of Ethnobiology, 2006; Bernard, 2011; American Anthropological Association, 2012). Due to the sensitive and confidential nature of ethnographic data, all primary ethnographic data (including field notes, field journals, photographs, archival materials, audio and video recordings, and transcriptions) are held in the custody and possession of the first author M.M.E.C., and stored and preserved indefinitely in secure archives. Ethnographic data may only be accessed by the core research group (M.M.E.C., F.A.A.G, and E.S.A.), in compliance with the ethical guidelines of the International Society of Ethnobiology (International Society of Ethnobiology, 2006; Pels et al., 2018). Furthermore, information and/or materials will not be released in any way in which participants can be personally recognized, unless authorised by the contributor (International Society of Ethnobiology, 2006).

We offered contributors copies of interview recordings. We committed to providing copies of research to the community, along with culturally appropriate materials for communicating results, such as videos or posters in Spanish, and asked for contributors' consent to reproduce or share images, audio, or video compiled in interviews or collected

during fieldwork (International Society of Ethnobiology, 2006). We also committed to recognizing the authorship materials collected during the course of fieldwork that were not generated by the research team, such as archival photos, documents or manuscripts, artwork, etc. (International Society of Ethnobiology, 2006).

REFERENCES

American Anthropological Association. 2012. *2012 Ethics Statement*.

Bernard HR. 2011. *Research Methods in Anthropology: Qualitative and Quantitative Approaches*. New York: AltaMira Press.

International Society of Ethnobiology. 2006. ISE Code of Ethics (with 2008 additions).

Pels P, Boog I, Henrike Florusbosch J, Kripe Z, Minter T, Postma M, Sleeboom-Faulkner M, Simpson B, Dilger H, Schönhuth M, von Poser A, Castillo RCA, Lederman R, Richards-Rissetto H. 2018. Data management in anthropology: the next phase in ethics governance? *Social Anthropology* 26:391–413. DOI: 10.1111/1469-8676.12526.

Sociedad Latinoamericana de Etnobiología. 2014. Código de ética para la investigación, la investigación-acción y la colaboración etnocientífica en América Latina. *Etnobiología* 12.

Wedemeyer-Strombel KR, Peterson MJ, Sanchez RN, Chavarría S, Valle M, Altamirano E, Gadea V, Sowards SK, Tweedie CE, Liles MJ. 2019. Engaging Fishers' Ecological Knowledge for Endangered Species Conservation: Four Advantages to Emphasizing Voice in Participatory Action Research. *Frontiers in Communication* 4:30. DOI: 10.3389/fcomm.2019.00030.
