# Supplementary material for: Quantifying local ecological knowledge to model historical abundance of long-lived, heavily-exploited fauna
Source: PeerJ. 2020 Jul 20;8:e9494. doi: 10.7717/peerj.9494 (PMC7377249; doi:10.7717/peerj.9494)
Supplement: Supplemental Information 3 [file peerj-08-9494-s003.docx]

| Analysis | Data | |
| --- | --- | --- |
|  | Qualitative | Quantitative |
| Qualitative | a) Discourse analysis, qualitative textual analysis | b) Search for meaning in results of quantitative processing (e.g., generating new questions based on GLM analysis) |
| Quantitative | c) Word counts; search for patterns, processes in quantitative textual analysis (Figures S2, S3) | d) Statistical and mathematical analysis of numerical data |
| Adapted from Bernard (2011) | | |

**Table S1:**

**Qualitative-Quantitative Data Analysis**
