# Supplementary material for: Quantifying local ecological knowledge to model historical abundance of long-lived, heavily-exploited fauna
Source: PeerJ. 2020 Jul 20;8:e9494. doi: 10.7717/peerj.9494 (PMC7377249; doi:10.7717/peerj.9494)
Supplement: Supplemental Information 4 [file peerj-08-9494-s004.docx]

**Table S2:**

**Number and type of interviews**

| **Years** | **Semi-structured** | **In-depth** | **Informal** | **Total** |
| --- | --- | --- | --- | --- |
| 2012-2013 | 22 | 20 | 186 | 228 |
| 2017-2018 | 17 | 11 | 122 | 150 |
| Total | 39 | 31 | 308 | 378 |
