# Supplementary material for: Quantifying local ecological knowledge to model historical abundance of long-lived, heavily-exploited fauna
Source: PeerJ. 2020 Jul 20;8:e9494. doi: 10.7717/peerj.9494 (PMC7377249; doi:10.7717/peerj.9494)
Supplement: Supplemental Information 5 [file peerj-08-9494-s005.docx]

| **Years** | **Working days** | **Journal pages** | **Transcription pages** | **Technical photographs** | **Archival material** | **Hours of audio recordings** | **Hours of video recordings** |
| --- | --- | --- | --- | --- | --- | --- | --- |
| 2012-2013 | 106 | 267 | 336 | 380 | 53 | 45 | 20 |
| 2017-2018 | 57 | 256 | 205 | 233 | 11 | 15 | 13 |
| Total | 163 | 523 | 541 | 613 | 64 | 60 | 33 |
|  | | | | | | | |

**Table S3:**

**Fieldwork inventory**
