# Supplementary material for: Quantifying local ecological knowledge to model historical abundance of long-lived, heavily-exploited fauna
Source: PeerJ. 2020 Jul 20;8:e9494. doi: 10.7717/peerj.9494 (PMC7377249; doi:10.7717/peerj.9494)
Supplement: Supplemental Information 6 — Date and study site are indicated at the beginning of the journal entry. Content is grouped in blocks of time. The approximate time of day and location, along with a general description of the activity and a cryptic indicator of the collaborator(s), are included in the heading of each block of time. Italics indicate categories adapted from the Outline of Cultural Materials (Murdock et al., 2008) at the beginning of each paragraph. Analysis, commentary, and cross-references are separated from observations with footnotes ([1]) at the end of each paragraph. Specific topics of interest are indexed using hashtags (#). […] indicates redacted segments. [file peerj-08-9494-s006.docx]

**Table S4:**

**Text box with example extract of a field journal entry (translated from Spanish and abbreviated). Bold type indicates numerical data.**

Date and study site are indicated at the beginning of the journal entry. Content is grouped in blocks of time. The approximate time of day and location, along with a general description of the activity and a cryptic indicator of the collaborator(s), are included in the heading of each block of time. Italics indicate categories adapted from the Outline of Cultural Materials (Murdock et al., 2008) at the beginning of each paragraph. Analysis, commentary, and cross-references are separated from observations with footnotes ([1]) at the end of each paragraph. Specific topics of interest are indexed using hashtags (#). […] indicates redacted segments.

| Saturday, July 29, 2017  Bahía de los Ángeles  […]  Informal interview with Mr. H  19:00-19:45, Mr. H’s House  *824.1,124*  We found Mr. H on the porch with his wife and one of his sons. "You’re late," he said, when we walked in. We told him that we’d gotten back from fishing at around 17:00, and we’d just now been able to make it over to see him. We told him we didn’t have many questions this time, and he said we could chat right then [1]. He asked if we’d interviewed Mr. B, and if he’d told us the story about the tiger shark […]  [1] This time we only had three follow-up questions from the semi-structured interview on 11/07/2017. All questions were about green turtle catch magnitude #methods.  *226.5.1, 441.1, 227*  In his first years fishing with nets, more or less between **1965** and **1967**, in a good night his crew could fill the vessel, but on a regular night they caught **4** or **5** green turtles in **two 60 fathom set-nets** #average_cpue #gear_characteristics. During the last years that he fished with nets, between **1968** and **1969** [1] "it was already a lot more scarce", he says, it **dropped by about half**. “About half as many fell in the net, maybe **2-3 per nigh**t” #average_cpue. However, "there were a lot of crews" #fishing_effort and they filled up to **4 tons in each truck** that was sent to market in Ensenada #commercial_volume. By those years, **it took up to a week to fill a truckload** [2] […]  [1] These were also the last years he fished for turtles. In 1970 he went to Ensenada to work in his family’s sea turtle restaurant (see journal 11/07/2019, interview transcription mrb_20170711.txt).  [2] This suggests reduced abundance. The trucks held **60-80 turtles**, at most **100** (**4 tonnes** would be approximately **80 turtles at 50 Kg each**). In the **early 60s**, some fishers report being able to **fill a truckload overnight or in a few days** (see #commercial_volume; Caldwell, 1962)  […] |
| --- |
