# Supplementary material for: Quantifying local ecological knowledge to model historical abundance of long-lived, heavily-exploited fauna
Source: PeerJ. 2020 Jul 20;8:e9494. doi: 10.7717/peerj.9494 (PMC7377249; doi:10.7717/peerj.9494)
Supplement: Supplemental Information 7 — The header includes the cryptic indicator for the collaborator and a list of their corresponding field journal entries and interview transcripts. Content includes summarized qualitative and quantitative information, quotes, follow-up notes, and commentary. [file peerj-08-9494-s007.docx]

**Table S5:**

**Text box with example of a fisher summary file (translated from Spanish)**

| Mr. D (mrd_20170726.txt, focusgroup_20170724.txt; Journals July 24-28, 2017)  Biographical data:   - Age: 65 (born 1952) - Arrived in BLA age 13 (1965) - Years in fishery: 1965-1971 - Worked in the Green turtle fishery from arrival until the early 1970s, when it became less profitable and he changed to the tourism industry.   Crew members: Mr. T, Mr. K  Buyer: Mr. E  Harpoon (1965):   - 1965-1966: “you could take as much as you wanted” with good weather - CPUE: vessel capacity (⁓1 ton) in good conditions - "That was when I started out, but later it got harder”   Set-nets (1966-1971):   - Low CPUE: 8-10 day trip for 4-5 turtles (final years with nets) - High CPUE: vessel capacity (⁓1 ton) in one night (occurred occasionally throughout career)   Fleet conditions:   - Vessels: mesquite-wood canoes throughout career - Capacity: ⁓1 ton (⁓20 turtles) - Motor: 10-25 horsepower (most years fishing with nets)   Notes:   - Scarcity started in the early 70s, when he stopped fishing - “With bigger motors, it started getting more scarce, because you could get places faster, just go out an come back” - Verify harpoon data with older crew members (very young, little experience) |
| --- |

The header includes the cryptic indicator for the collaborator and a list of their corresponding field journal entries and interview transcripts. Content includes summarised qualitative and quantitative information, quotes, follow-up notes, and commentary.
