# Supplementary figures and images for: Quantifying local ecological knowledge to model historical abundance of long-lived, heavily-exploited fauna
Source: PeerJ. 2020 Jul 20;8:e9494. doi: 10.7717/peerj.9494 (PMC7377249; doi:10.7717/peerj.9494)

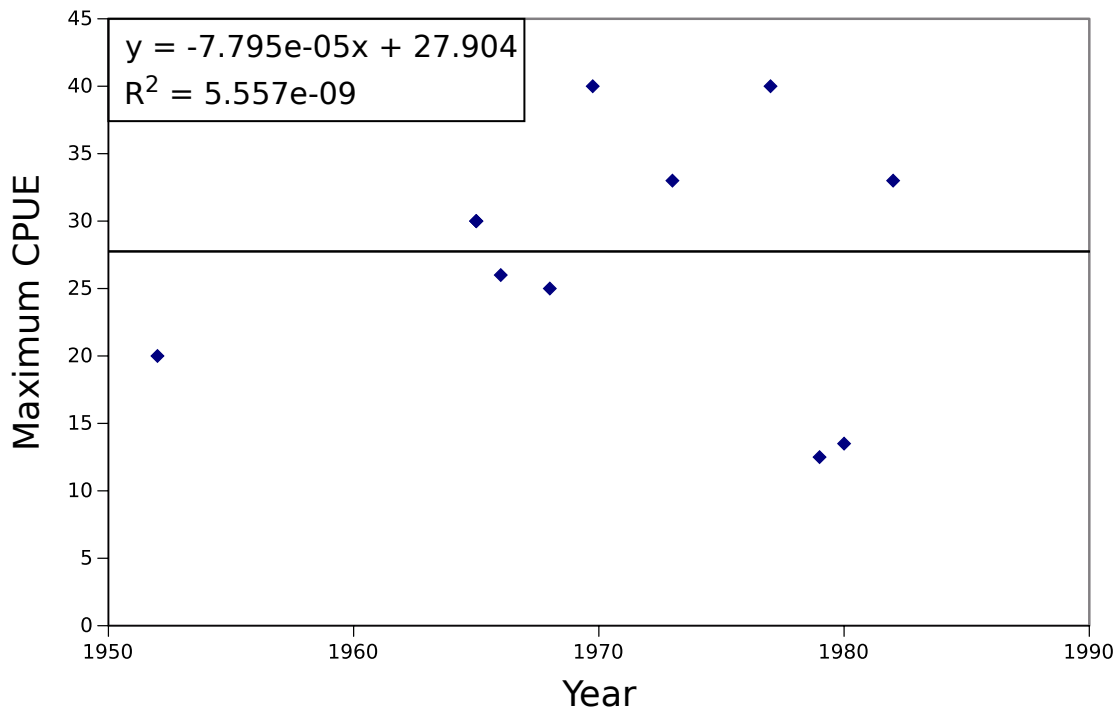

Supplement: Supplemental Information 8 [file peerj-08-9494-s008.pdf]

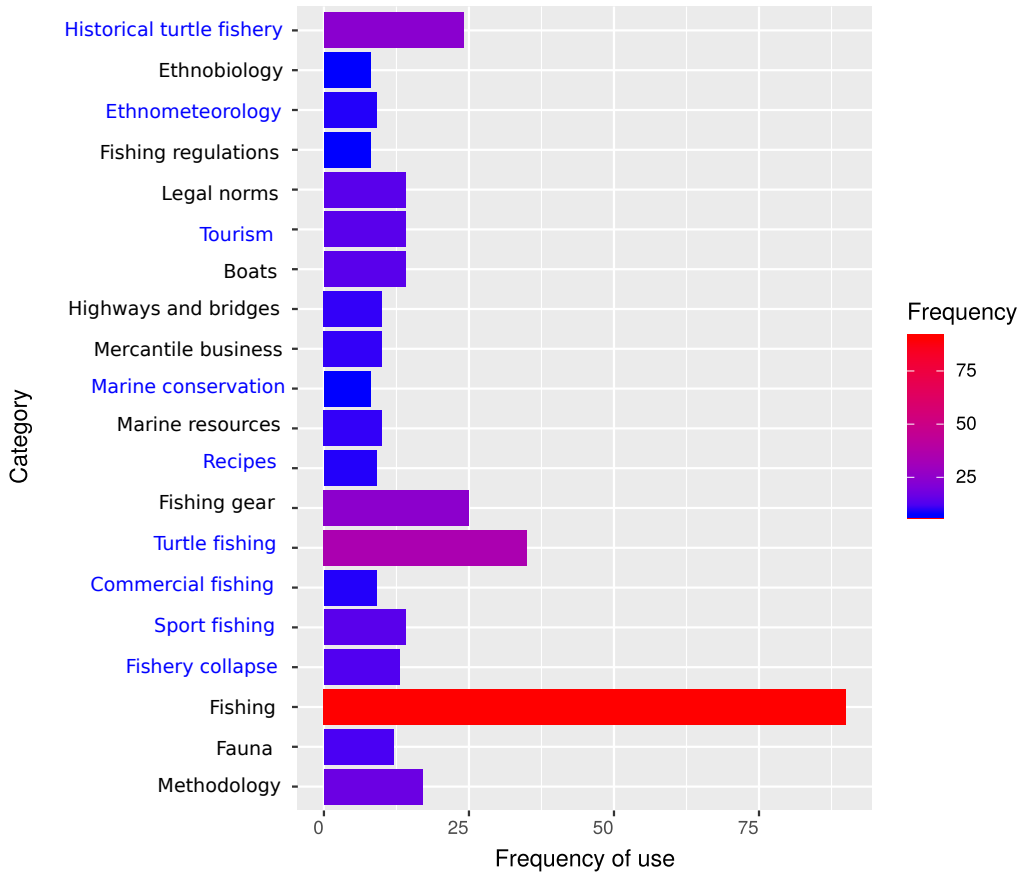

Supplement: Supplemental Information 9 — Cultural material codes used most frequently in field journals. Blue text indicates customized codes adapted from Murdock et al. (2008). [file peerj-08-9494-s009.pdf]

CPUE (turtles/mi<sup>2</sup>)

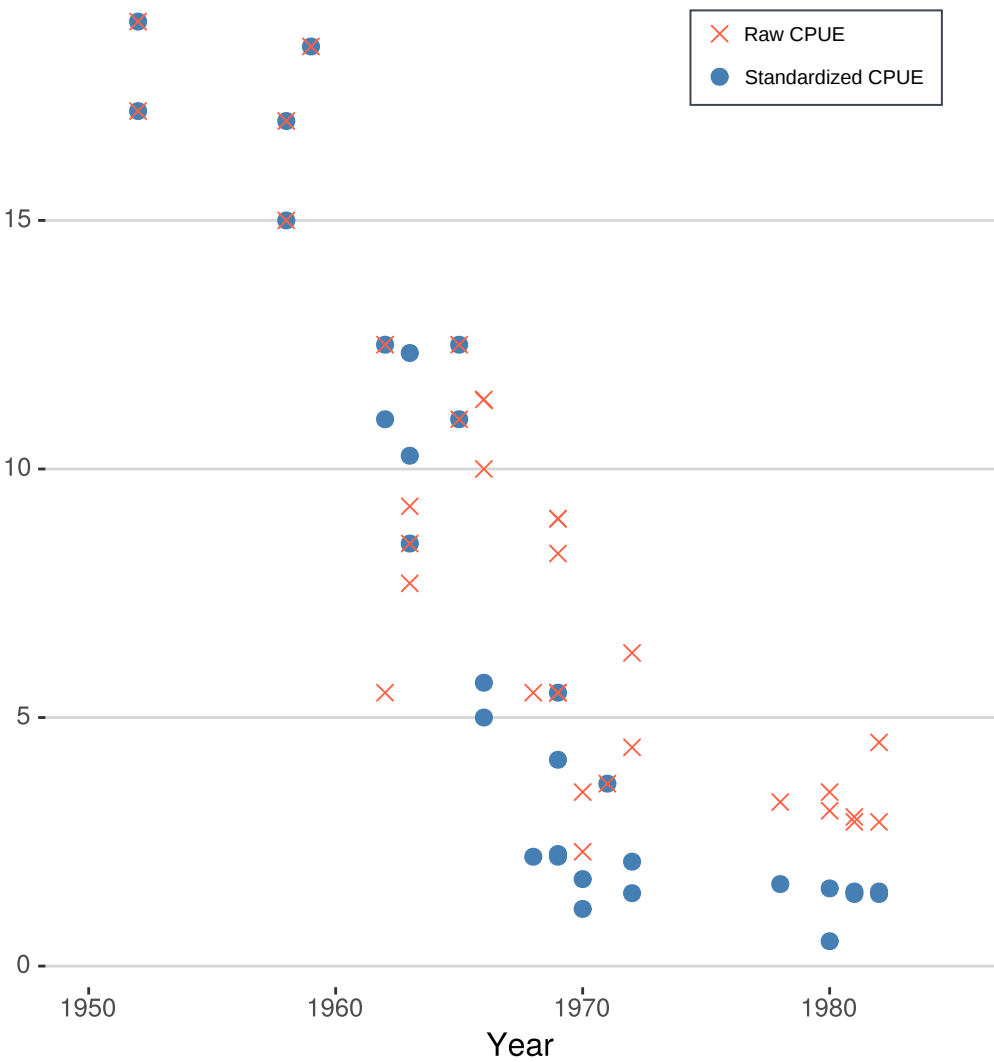

Supplement: Supplemental Information 11 — Data points represent raw CPUE (red crosses) and standardized CPUE (blue circles). Data points for years with multiple CPUE values are included. [file peerj-08-9494-s011.pdf]

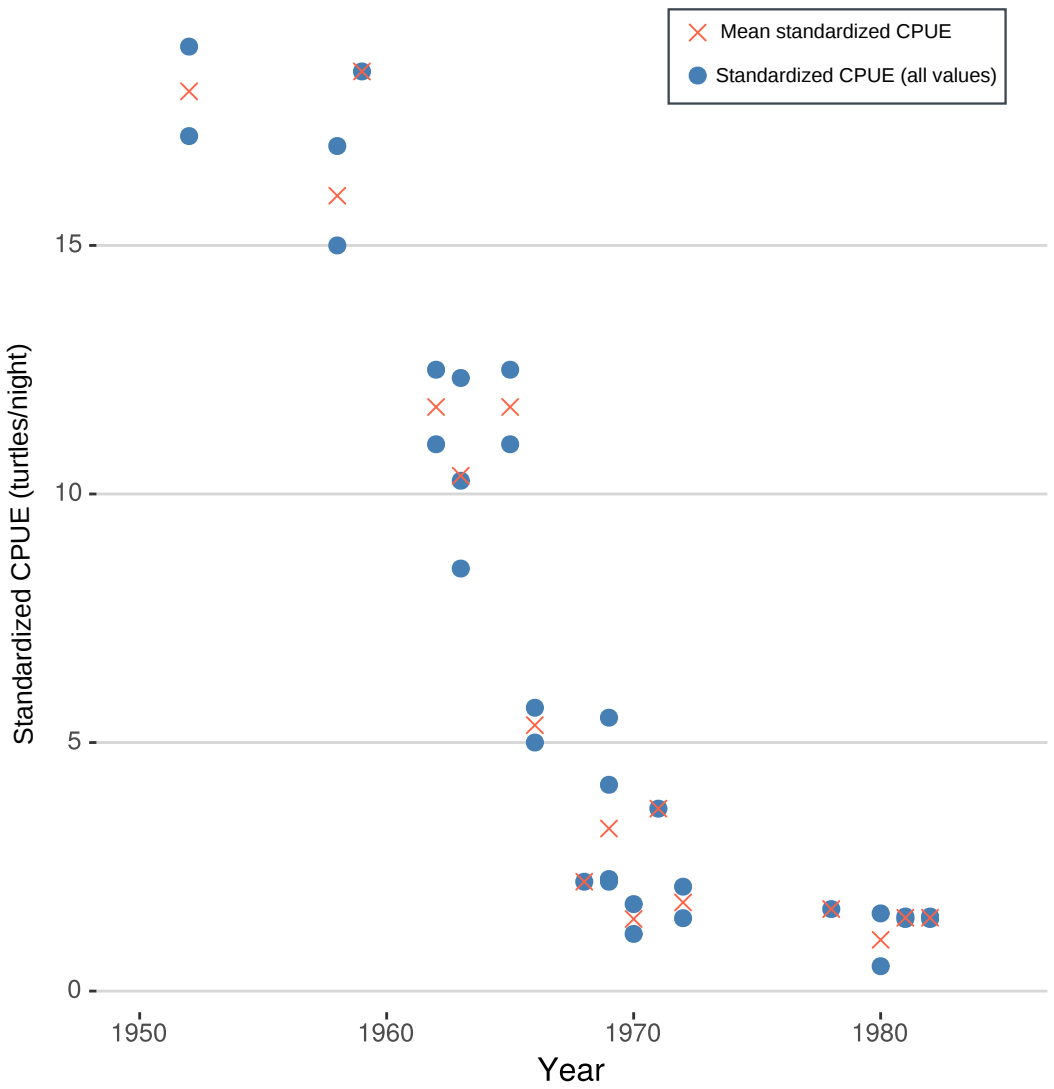

Supplement: Supplemental Information 12 — Blue circles represent all standardized CPUE values, including years with multiple data points. Red crosses represent the mean standardized CPUE values for a given year. Mean, standardized CPUE values (red crosses) were used for final analyses. [file peerj-08-9494-s012.pdf]

z-scores for CPUE and annual landings

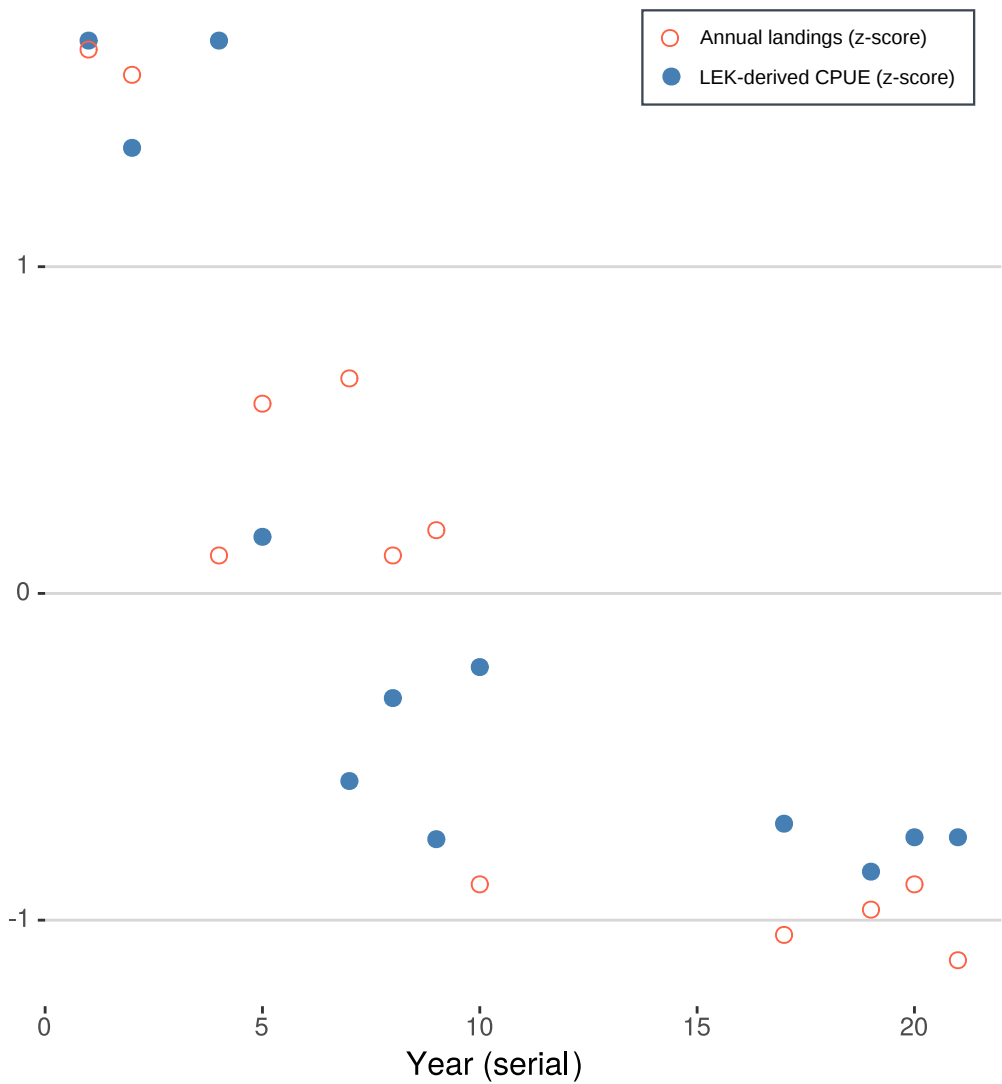

Supplement: Supplemental Information 13 — Z-scores for LEK-derived CPUE (blue circles) and annual landings from fisheries statistics available for 1962-1982 for Bahía de los Ángeles (red circles) (Márquez in Seminoff et al., 2008) [file peerj-08-9494-s013.pdf]

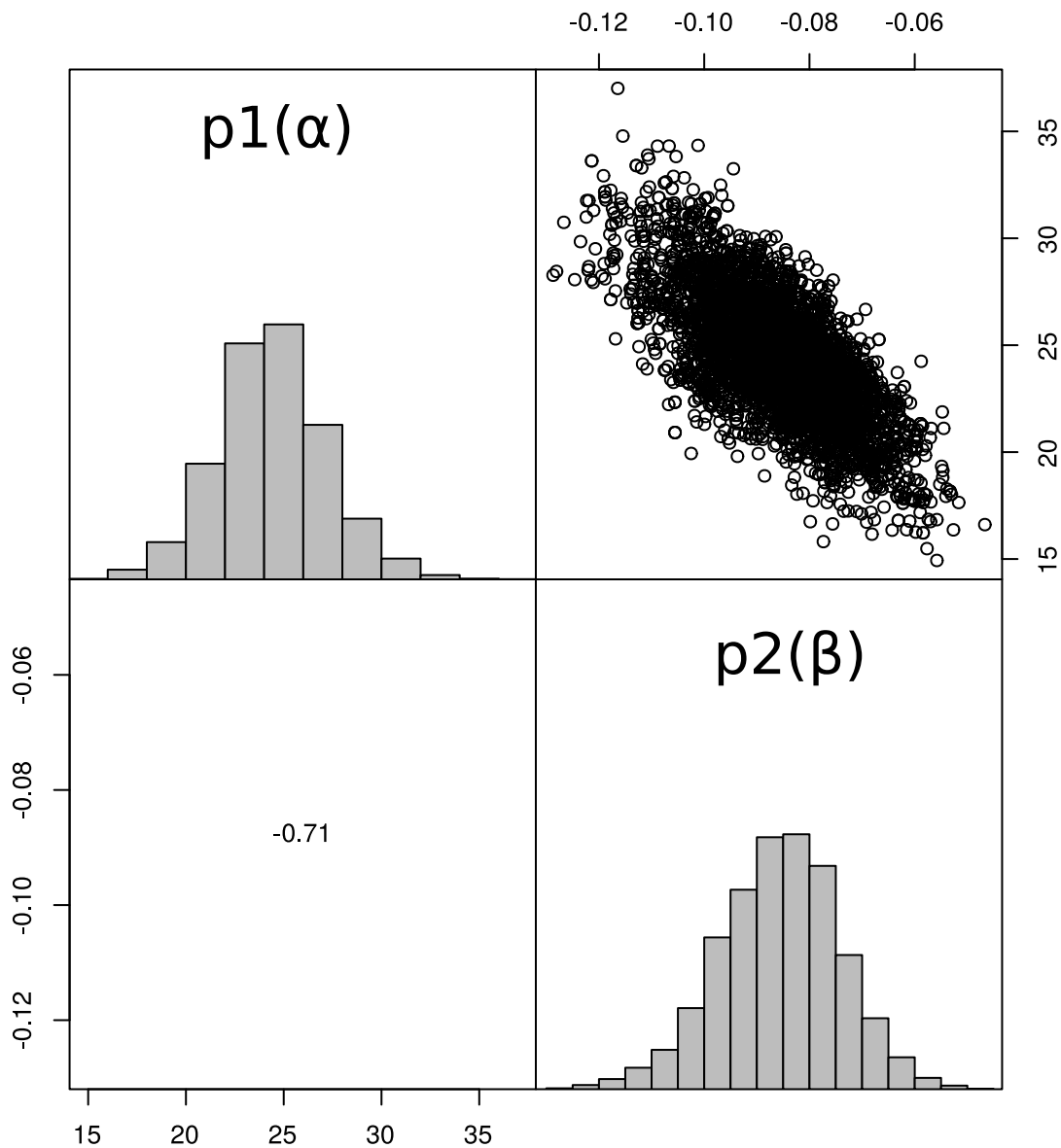

Supplement: Supplemental Information 14 — Pairs plot of the MCMC sensitivity analysis of for both parameters of the nonlinear regression model with best fit (eqn. 2, Figure 6 in main text). The upper panel shows the pairwise relationship of both parameters, the lower panel shows correlation coefficients, and the diagonal shows marginal distribution for each parameter represented by a histogram. [file peerj-08-9494-s014.pdf]

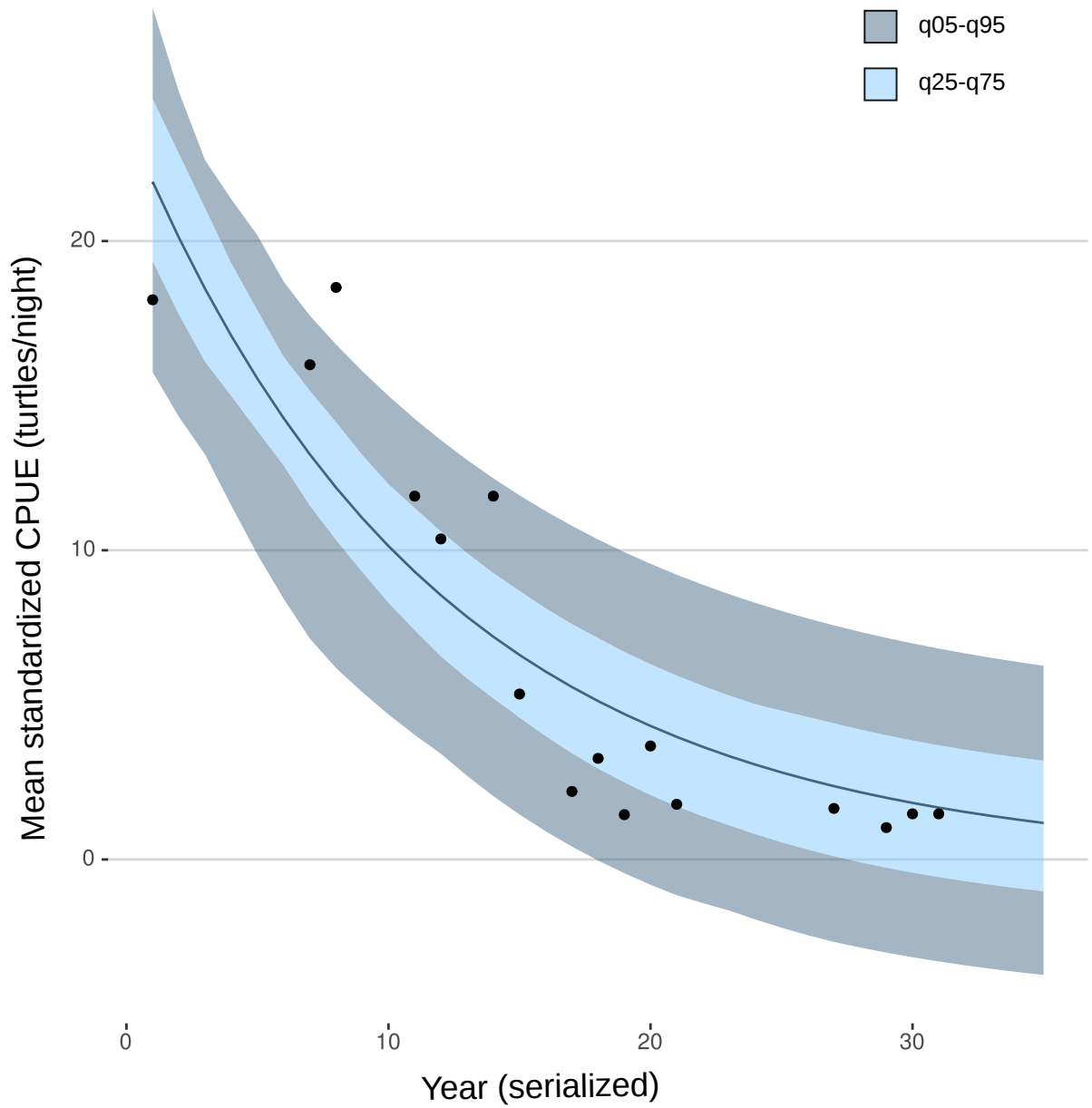

Supplement: Supplemental Information 15 — Posterior predictive distribution of the nonlinear regressios model with best fit (eqn. 2, Figure 6 in main text) obtained from a randomly selected subset of 100 parameter combinations from the MCMC chain and randomly distributed noise to estimate measurement error. Black dots represent mean standardized catch-per-unit-effort values for C. mydas en Bahía de los Ángeles. [file peerj-08-9494-s015.pdf]
