# Supplementary material for: Quantifying local ecological knowledge to model historical abundance of long-lived, heavily-exploited fauna
Source: PeerJ. 2020 Jul 20;8:e9494. doi: 10.7717/peerj.9494 (PMC7377249; doi:10.7717/peerj.9494)
Supplement: Supplemental Information 17 [file peerj-08-9494-s017.docx]

**COREQ Checklist**

**Manuscript:** Quantifying Local Ecological Knowledge to Model Past Abundance of Long-lived, Heavily-Exploited Fauna

**Journal**: PeerJ

**Authors:** Early-Capistrán, Michelle-María, Solana-Arellano, Elena, Abreu-Grobois, F. Alberto, Narchi, Nemer E., Garibay-Melo, Gerardo, Seminoff, Jeffrey A., Koch, Volker, Sáenz-Arroyo, Andrea

| **No. Item** | **Guide questions/description** | **Reported on Page #** |
| --- | --- | --- |
| **Domain 1: Research team and reﬂexivity** |  |  |
| *Personal Characteristics* |  |  |
| 1. Inter viewer/facilitator | M.M.E.C. and G.G.M. conducted all interviews | Methods |
| 2. Credentials | The first author and primary researcher, M.M.E.C., is a PhD candidate in Ocean Science, M.Sc. in Ocean Science, and B.A. in Cultural Anthropology. G.G.M. is an MSc in Drylands Ecosystem Management and a B.A. in Philosophy. | N/A |
| 3. Occupation | At the time fieldwork was conducted, M.M.E.C. was a PhD student and G.G.M. was an MSc Student. | N/A |
| 4. Gender | M.M.E.C. is a woman, G.G.M. is a man | N/A |
| 5. Experience and training | M.M.E.C. is a cultural anthropologist who has conducted ethnographic research in Latin America since 2002, and has worked with fishing communities in the Mexican Pacific since 2007. G.G.M. has a B.A. in Philosophy and has conducted ethnographic fieldwork in fishing communities in Northwest Mexico since 2012. | N/A |
| *Relationship with participants* |  |  |
| 6. Relationship established | Both M.M.E.C. and G.G.M. have conducted ethnographic research in the study area since 2012, and had worked previously with ~85% of study participants. | N/A |
| 7. Participant knowledge of the interviewer | The research team obtained permission from local authorities, and clearly disclosed the aims and objectives of the research, as well as institutional backing and funding sources to participants. All participation was voluntary, and we clearly communicated to participants the right to reserve responses to any questions. We also gained informed, verbal consent to carry out or record interviews, and to take photographs or produce audio or video recordings | Methods;  Supporting Information: Article S1, Article S2 |
| 8. Interviewer characteristics | Interviewers reported the purpose and aims of the research to participants. | Supporting Information, Article S1 |
| **Domain 2: study design** |  |  |
| *Theoretical framework* |  |  |
| 9. Methodological orientation and Theory | Ethnography was the primary methodological approach for this research, in conjunction with elements of grounded theory. | Methods |
| *Participant selection* |  |  |
|  | We defined three social groups within the community and documented their knowledge: (i) Fishers who participated in the legal green turtle fishery before 1990 (henceforth, turtle fishers) constituted the target population and provided the majority of specialized LEK related to human-turtle interaction; (ii) Key local collaborators —defined as community members with expertise in particular topics— provided important complementary and contextual information; and (iii) members of the community at large (henceforth, community members), including fishers’ families, green turtle merchants, local authorities, commercial and sport fishers, and conservation workers, provided complementary data.   - Turtle fishers: Deliberate hierarchical sampling - Key local collaborators: purposive and respondent-driven sampling - Community members: cluster sampling and self-selection | Methods |
|  | Face to face | N/A |
| 10. Sampling | - Turtle fishers: n=16 (94% of the statistical population) - Key local collaborators: n=7 - Community members: n=48 (~8% of the community’s population) | Methods |
| 11. Method of approach | 1 person chose not to participate | Methods |
| *Setting* |  |  |
| 14. Setting of data collection | Participant observation and informal interviews were conducted at several locations in the community and at sea with fishers. Semi-structured interviews, in-depth interviews, and focus groups were conducted primarily in fishers’ homes or in public spaces in the community. | N/A |
| 15. Presence of non-participants | Family members and/or friends were present during the majority of semi-structured and/or in-depth interviews (~85%). | N/A |
| 16. Description of sample | - Turtle fishers are a small group of the oldest fishers in the community, between 55 and 85 years of age (n=16). All fishers in the population and sample were men. - Key local collaborators were primarily older (>63, 71%). 43% were women and 57% were men - Community members were split relatively evenly among genders (42% women, 58% men). Ages ranged from 18-93, with young (18-39, 35%), middle-aged (40-62, 37%), and older (>63, 28%) participants. The group included both long-term residents (89%) and short-term residents (11%) | Methods |
| *Data collection* |  |  |
| 17. Interview guide | Flexible topic guides were used for semi-structured and in-depth interviews. Guides covered 5 main areas: (1) biographical profile and career history; (2) sea turtle consumption and commerce; (3) trends in sea turtle captures and sizes; (4) spatial distribution of sea turtle fishing; and (5) fishing effort and technology.  Questions were piloted with local fishers outside the target population (n=2), and were constantly refined to ensure that they were locally contextualised and elicited meaningful answers. | Methods, Box 1 |
| 18. Repeat interviews | Key local collaborators and expert LEK holders were interviewed repeatedly. | Methods |
| 19. Audio/visual recording | Semi-structured interviews, in-depth interviews and focus groups were recorded in audio and/or video with participants’ informed verbal consent | Methods |
| 20. Field notes | Field notes were recorded continuously during the course of field work. | Methods |
| 21. Duration | Interviews varied in duration from 5-10 minutes in the case of the shortest informal interviews, to over 3 hours in the case of the longest in-depth interviews. On average, semi-structured interviews, in-depth interviews, and focus groups had duration of 60-90 minutes. | N/A |
| 22. Data saturation | Field work was conducted until reaching topical saturation, thematic saturation, and data saturation. | Methods |
| 23. Transcripts returned | Transcripts were not returned to participants. However, participants were offered copies of interview recordings. | Supporting Information, Article S1 |
| **Domain 3: analysis and ﬁndings** |  |  |
| *Data analysis* |  |  |
| 24. Number of data coders | M.M.E.C. coded the data | N/A |
| 25. Description of the coding tree | We did not use a coding tree. Cultural material codes were used to categorise ethnographic data, with custom codes for specific topics. Text entries were indexed using hashtags (#) to mark relevant topics. | Methods;  Supporting Information, Article S1 |
| 26. Derivation of themes | Themes were identified through background research and derived from data in the field and through the iterative process described throughout the Methods. | Methods |
| 27. Software | To ensure cross-platform compatibility and longevity, field notes, journals and transcriptions were compiled in .txt format (SublimeText) and quantitative data were stored in .csv format (R 3.4). Analyses were carried out in R 3.4, LABFit 7.2.49, and QtiPlot 0.9.9.7. | Methods |
| 28. Participant checking | We integrated feedback from community members by sharing preliminary results through narrative description. | Methods |
| *Reporting* |  |  |
| 29. Quotations presented | Quotations are included as examples in Figure 4. Collaborators are identified with a cryptic indicator. | Methods, Figure 4 |
| 30. Data and ﬁndings consistent | Yes | N/A |
| 31. Clarity of major themes | Yes. Major themes are discussed in the manuscript. | Methods, Results, Discussion |
| 32. Clarity of minor themes | Minor themes are discussed in the manuscript and presented extensively in the Supporting Information | Methods, Results, Discussion;  Supporting Information, Article S1 |
